# Peptide location fingerprinting reveals modification‐associated biomarker candidates of ageing in human tissue proteomes

**DOI:** 10.1111/acel.13355

**Published:** 2021-04-08

**Authors:** Matiss Ozols, Alexander Eckersley, Kieran T. Mellody, Venkatesh Mallikarjun, Stacey Warwood, Ronan O'Cualain, David Knight, Rachel E. B. Watson, Christopher E. M. Griffiths, Joe Swift, Michael J. Sherratt

**Affiliations:** ^1^ Division of Cell Matrix Biology & Regenerative Medicine The University of Manchester Manchester UK; ^2^ Division of Musculoskeletal & Dermatological Sciences The University of Manchester Manchester UK; ^3^ Wellcome Centre for Cell‐Matrix Research The University of Manchester Manchester UK; ^4^ Biological Mass Spectrometry Core Research Facility School of Biological Sciences Faculty of Biology, Medicine and Health The University of Manchester Manchester UK; ^5^ NIHR Manchester Biomedical Research Centre Central Manchester University Hospitals NHS Foundation Trust Manchester Academic Health Science Centre Manchester UK

**Keywords:** ageing, biomarkers, mass spectrometry, peptide location fingerprinting, photoageing, proteomics, skin, tendon

## Abstract

Although dysfunctional protein homeostasis (proteostasis) is a key factor in many age‐related diseases, the untargeted identification of structurally modified proteins remains challenging. Peptide location fingerprinting is a proteomic analysis technique capable of identifying structural modification‐associated differences in mass spectrometry (MS) data sets of complex biological samples. A new webtool (Manchester Peptide Location Fingerprinter), applied to photoaged and intrinsically aged skin proteomes, can relatively quantify peptides and map statistically significant differences to regions within protein structures. New photoageing biomarker candidates were identified in multiple pathways including extracellular matrix organisation (collagens and proteoglycans), protein synthesis and folding (ribosomal proteins and TRiC complex subunits), cornification (keratins) and hemidesmosome assembly (plectin and integrin α6β4). Crucially, peptide location fingerprinting uniquely identified 120 protein biomarker candidates in the dermis and 71 in the epidermis which were modified as a consequence of photoageing but did not differ significantly in relative abundance (measured by MS1 ion intensity). By applying peptide location fingerprinting to published MS data sets, (identifying biomarker candidates including collagen V and versican in ageing tendon) we demonstrate the potential of the MPLF webtool for biomarker discovery.

## Graphical abstract

Peptide location fingerprinting is a proteomic mass spectrometry tool capable of detecting localised statistically significant changes in peptide yield along the structures of proteins in complex, whole tissue lysates. In this study, peptide location fingerprinting revealed novel biomarker candidates of skin photoageing undetectable by conventional relative quantification.

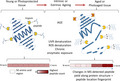

## 1 INTRODUCTION

Loss of proteostasis is a key feature of many age‐associated diseases (Hipp et al., 2019). In human skin, ageing induces remodelling of key structural proteins which impacts on appearance and function. This loss of proteostasis is particularly evident in sites exposed to ultraviolet radiation (UVR) (Flament et al., 2013) where photoageing differentially affects the keratinocyte‐rich epidermis and the underlying extracellular matrix (ECM)‐rich dermis (Langton et al., 2019). Within the dermis, some ECM macromolecular assemblies, such as collagen I and elastic fibres, are long‐lived with biological half‐lives spanning decades (Shapiro et al., 1991; Sivan et al., 2008). These abundant structural proteins are thought to accumulate photoageing‐induced damage over time (Naylor et al., 2011) as a consequence of chronic UVR exposure (predominantly UVA) (Eckersley et al., 2020; Hibbert et al., 2015; Sherratt et al., 2010), UVR‐induced reactive oxygen species (ROS) (Hibbert et al., 2019; Sander et al., 2002) and elevated protease activity (Brennan et al., 2003). It is not known, however, whether proteins are similarly modified as a consequence of ageing in disparate matrisomes. In contrast to the dermis, epidermal cells and biomolecules are exposed to higher energy UVB, leading to the progressive accumulation of DNA damage (Cadet et al., 2005) and modulation in gene expression and potential loss of function (Yaar & Gilchrest, 2001). Due to higher protein turnover, it is likely that damage manifests primarily in the epidermis as changes in the ability of cells to synthesise functional proteins (Cho et al., 2018). Due to the biological complexity of skin and these varied mechanisms, the photoageing process creates a spectrum of modifications to proteins which are challenging to track using a single methodology. It is therefore necessary to develop novel methods for the identification of these proteins.

The characterisation of tissue proteostasis and discovery of novel ageing biomarker candidates is crucial for deciphering the effects of ageing and for the identification of new therapeutic targets (Johnson et al., 2020). Label‐free proteomic liquid chromatography–tandem mass spectrometry (LC‐MS/MS) is a powerful analytical technique used traditionally for the identification and relative quantification of proteins within complex tissue extracts. The technique has enabled the identification of disease and ageing biomarkers based on their relative abundance (Newton et al., 2019); however, the detection of abundance‐independent, modification‐associated biomarkers in complex protein mixtures remains challenging.

**FIGURE 1:**
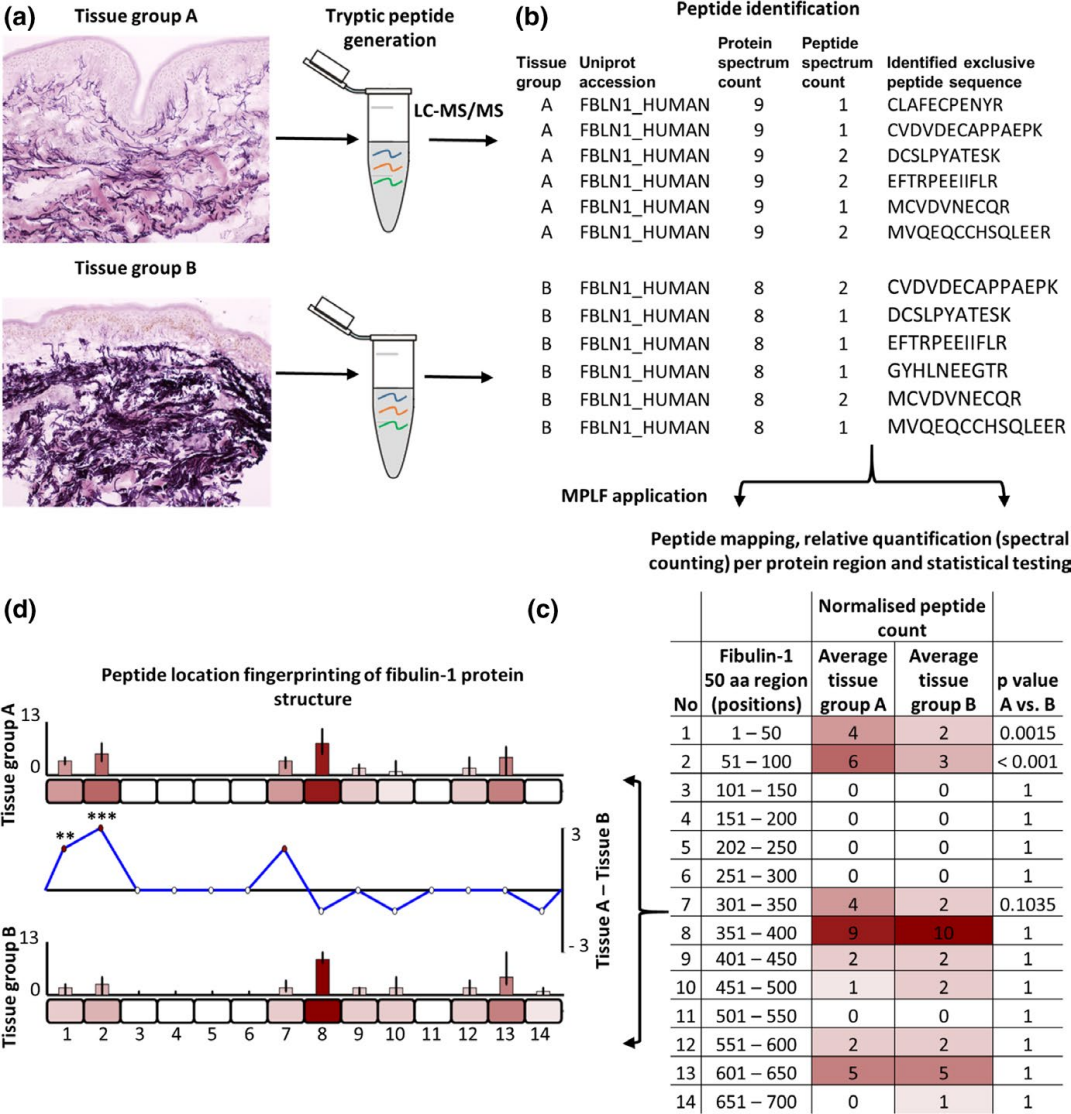
Identification of regional peptide yield differences within proteins using peptide location fingerprinting. Proteins are first extracted from tissues of interest using study‐specific protocols and trypsin digested (a). Post LC‐MS/MS, exclusive tryptic peptide sequences are identified and counted (spectral counting, i.e. peptide spectrum matches; PSMs) per tissue sample. Fibulin‐1 is shown here as an exemplar (b). Peptide count reports are uploaded to MPLF (c) where protein structures are divided either into user‐defined amino acid (aa) step sizes (e.g. 50 aa shown here) or into step sizes corresponding to domains, repeats or regions pre‐defined by the UniProt database. Peptides and their counts are then mapped to their protein regions and summed. Sample‐specific regional counts are normalised across the experiment based on the median protein spectrum count, averaged per tissue group and statistically compared (Bonferroni‐corrected repeated measures ANOVA). This analysis is then visualised using representative aa‐scale schematics of each protein (d). Average peptide counts are heat mapped to their corresponding region for comparison between groups (bar graphs). Regional, average peptide counts in one tissue group are subtracted from the counts of the other to show regional differences in peptide yield (line graph) with statistical significances indicated (** ≤0.01, *** ≤0.001)

Mass spectrometry analysis of post‐translational modifications is predominantly tailored towards residue specificity, (e.g. phosphorylation and methylation) which is well suited to the study of intracellular signalling and metabolism (Ke et al., 2016). However, this level of site‐specific analysis in unsuitable for photoageing and ageing long‐lived ECM proteins, since residues can be variably and sporadically modified across structures over time. In order to address the need for an approach capable of identifying proteins with these intermittent and highly variable photoageing‐related modifications, we have developed the Manchester Peptide Location Fingerprinter (MPLF) webtool which can detect average changes in peptide yield along protein structures (Eckersley et al., 2018, 2020). MPLF is an LC‐MS/MS proteomic analysis tool which implements peptide location fingerprinting in which tryptic peptide spectral counts are mapped to protein regions (specified by the user, or as defined in the UniProt database (Consortium, 2016)), relatively quantified per region and statistically tested between groups (Figure 1).

In standard proteomics, proteins are exposed to enzymes (e.g. trypsin), generating peptides whose sequence identities and quantities are then measured by LC‐MS/MS. Peptide location fingerprinting exploits the partial digestion of proteins due to their differing solubilities, stabilities and structure‐related enzyme susceptibilities. We have previously shown that the digestibility of structural regions within proteins varies between tissues (Eckersley et al., 2018) and as a result of UVR‐induced damage (Eckersley et al., 2020). Therefore, by mapping and quantifying peptides within specific protein regions, peptide location fingerprinting enables the detection of statistically robust regional differences as a consequence of modifications to protein structure (though not the specific modification site). This is particularly true for the highly insoluble ECM proteins and supramolecular assemblies.

In a proof of concept study using peptide location fingerprinting, we have previously shown *in vitro* that regional changes in 3°/4° structures by UVR‐induced damage modifications to protein can be detected in cell‐derived suspensions of isolated ECM assemblies (Eckersley et al., 2020). In an earlier study using the same approach, we also revealed that long‐lived macromolecular ECM assemblies exhibit intertissue structural diversity (Eckersley et al., 2018). Here, we show that this same method can be used as a surveying tool to detect statistically significant, photoageing‐specific, structural differences in proteins within human epidermal and dermal proteomes. In addition to this, MPLF was further used to analyse a previously published human ageing tendon data set (Hakimi et al., 2017) demonstrating the webtool's capability in evaluating existing LC‐MS/MS data. This enables the identification of potentially novel biomarkers and pathways which are independent of protein abundance when compared to traditional relative quantification by proteomic MS.

## 2 RESULTS AND DISCUSSION

### 2.1 Peptide location fingerprinting reveals regional fluctuations in peptide yield within the structures of proteins from photoaged skin

Prior to LC‐MS/MS, we confirmed that donor forearm skin was severely photoaged compared with matched, intrinsically aged buttock skin. Skin sections from extensor forearm skin exhibited clear hallmarks of photoageing (Figure [Link], [Link]) including epidermal thinning, disruption of elastic fibre architecture (Watson et al., 2014) and solar elastosis (Figure [Link], [Link]).

**FIGURE 2:**
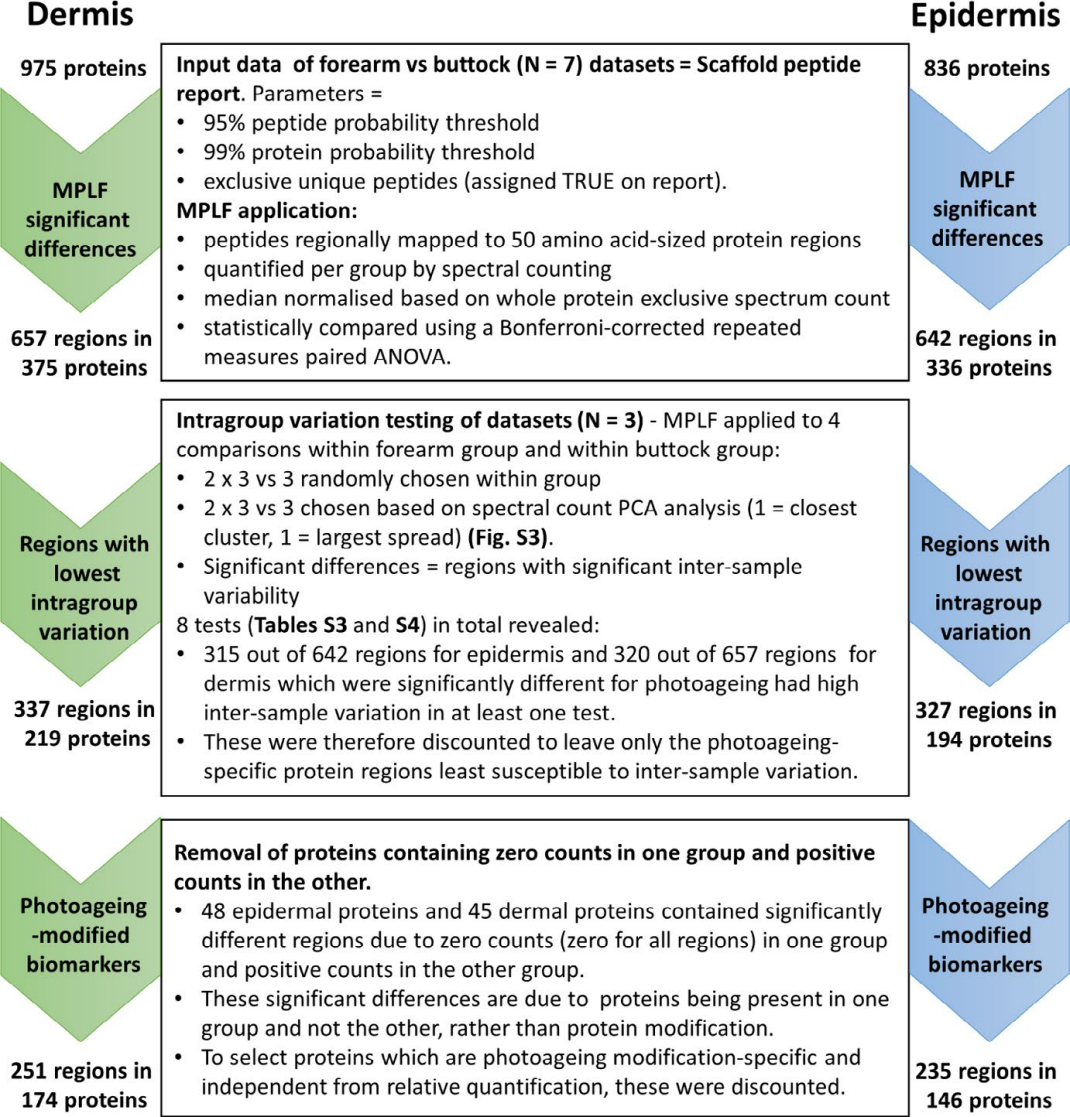
Identification of photoageing‐modified biomarker candidates in human skin by filtering significant regional differences detected using the MPLF webtool. MPLF was applied to dermal and epidermal data sets to identify 50 aa‐sized protein regions with significant differences in peptide yield between forearm and buttock samples. Once identified, significantly different regions were tested for intragroup variation (Tables S3 and S4). Regions which exhibited significant intragroup variation and proteins which were only present in one group (forearm or buttock) were excluded to leave only those which were photoageing‐specific. The remaining regions and their respective proteins were considered as indicators of structural modification, and therefore as new biomarker candidates of photoageing

As the epidermis and dermis contain distinct molecular and cellular populations, we separated these layers and analysed them independently with LC‐MS/MS. A total of 51,895 tryptic peptide sequences corresponding to 975 proteins (Table S1) were detected across all dermal samples and 45,868 (836 proteins) across epidermal samples (Table S2). Principal component analysis (PCA) of peptide spectral counts showed a clear separation of forearm and buttock data into distinct clusters (Figure [Link], [Link]) highlighting the global difference between photoaged and intrinsically aged skin. Peptide location fingerprinting was then applied using the MPLF webtool. Proteins were segmented into 50 amino acid (aa) sized regions and peptide sequences then mapped and relatively quantified within each (spectral count per region) and statistically compared between forearm and buttock groups (Figure 2).

The MPLF webtool allows protein structures to be divided either into equal regions, using step sizes of 20, 40, 50, 80 or 100 aa, or into regions corresponding to domains or repeats as pre‐defined by the UniProt database. In this study, a segment size of 50 aa was deemed optimal as it: 1) enabled analysis across the entire protein structure (not achievable by using conserved domains and regions), 2) standardised the size of these regions for comparison across all proteins and 3) was sufficiently large to achieve a peptide count high enough for accurate statistical comparison within proteins, with sufficient peptide coverage, but small enough to resolve changes across the structures of small proteins.

Using the MPLF webtool, peptide location fingerprinting identified 657 protein regions in dermis and 642 regions in epidermis (corresponding to 375 and 336 proteins, respectively) which had significant fluctuations in peptide yield between forearm and buttock samples (Figure 2). Of these protein regions, 337 for dermis and 327 for epidermis were least susceptible to intragroup variation (Tables S3 and S4) and therefore most photoageing‐specific (corresponding to 219 and 194 proteins, respectively) (Figure 2). Lastly, to identify regional differences that are most independent from protein presence, proteins which were present in only forearm or buttock group were discounted. This left a total of 251 regions within 174 proteins in dermis (Table S5) and 235 regions within 146 proteins in epidermis (Table S6) to be considered as biomarker candidates susceptible to photoageing‐related modifications (Figure 2).

**FIGURE 3:**
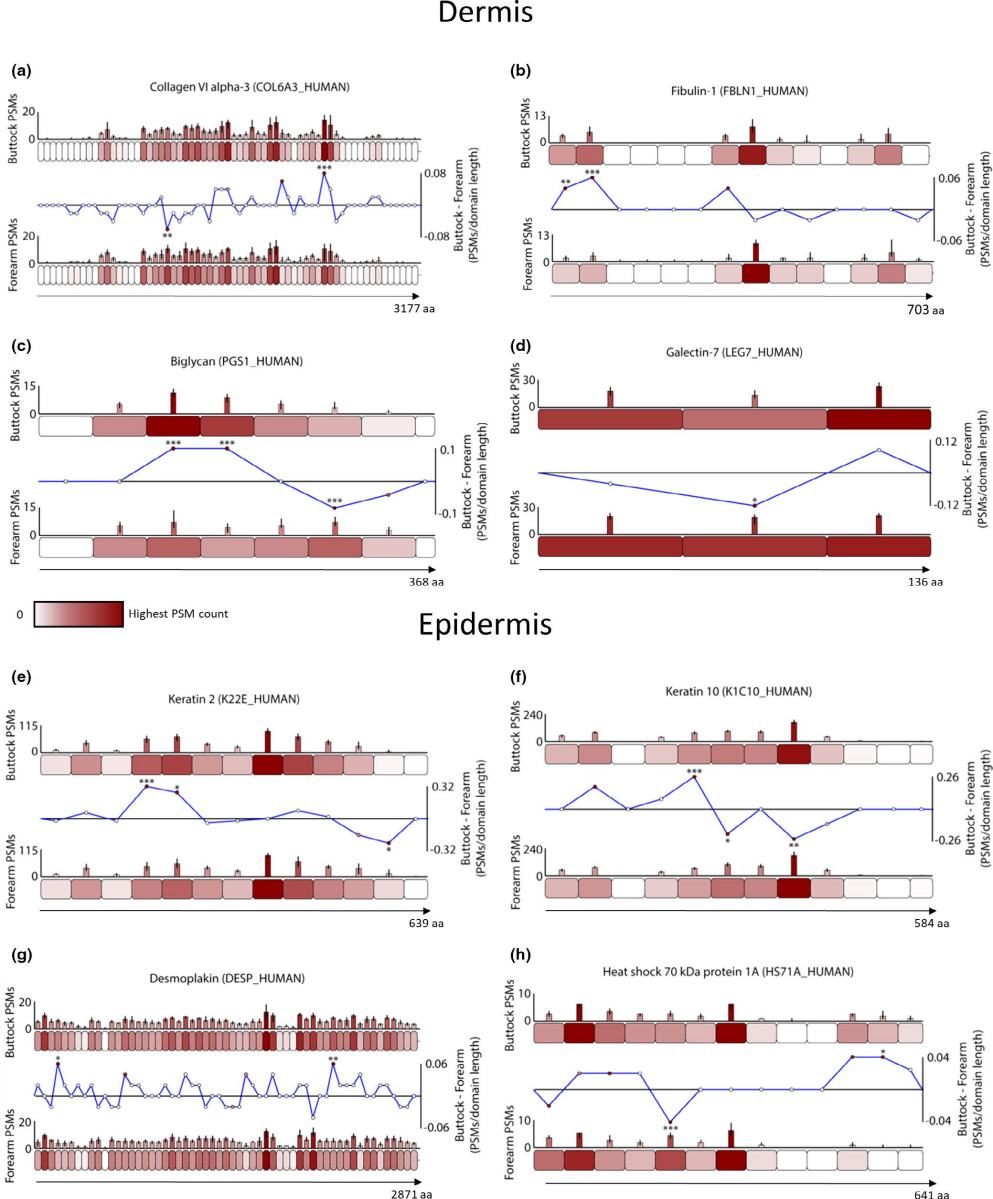
Exemplar skin biomarker candidates exhibit regional differences in peptide yield due to photoageing‐specific modifications. Proteins were segmented into 50 aa‐sized step regions with average peptide counts (PSMs; *N* = 7) heat mapped to each step and compared between forearm and buttock (bar graphs = average PSMs, error bars = SD). Average peptide counts corresponding to each forearm protein step were subtracted from the counts of their corresponding buttock protein step and divided by the aa sequence length of that step to reveal regional differences in peptide yield (line graphs). Multiple proteins within the dermis (a–d) and epidermis (e–h) exhibited statistically significant regional differences between forearm and buttock (* ≤0.05, ** ≤0.01, *** ≤0.001; Bonferroni‐corrected repeated measures paired ANOVA). In the dermis, one region in the N‐terminal half of collagen VI alpha‐3 had significantly lower peptide counts in buttock samples than in forearm, whereas another region in the C‐terminal half of the protein had significantly higher (a). Two N‐terminal regions within fibulin‐1 had significantly higher peptide counts in buttock than in forearm (b). Biglycan had two regions near the central portion of the protein with significantly higher peptide counts in buttock than in forearm and another region on the C‐terminal side with significantly lower (c). A central region in galectin‐7 yielded significantly lower peptides in buttock samples than in forearm (d). In epidermis, two N‐terminal sided regions in K2 (e) and one in K10 (f) had significantly higher peptide counts in buttock than in forearm whereas one region near the C‐terminal end of K2 and two near the protein centre of K10 had significantly lower counts in buttock than forearm. In desmoplakin (g) one N‐terminal region and another near the C‐terminus yielded significantly higher peptides in buttock than in forearm. Lastly, HSP70 exhibited one N‐terminal sided region with significantly lower peptide counts in buttock than in forearm and another C‐terminal region with significantly higher (h)

Of the protein biomarker candidates identified with local molecular differences, eight exemplar proteins that play key functional roles in skin are displayed in Figure 3 (interleaved graphs: Figure [Link], [Link]): collagen VI alpha‐3, fibulin‐1, biglycan and galectin‐7 from the dermis and keratins (K)‐2 and −10, desmoplakin and heat shock protein (HSP) 70 from the epidermis. The differences in peptide yield within the structural regions of these proteins are resultant from their altered exposure to trypsin as a consequence of photoageing. This may be due to 1) changes in protein synthesis, 2) allosteric‐dependent differences in protein folding (where UVR‐related modifications along the structure may lead to alterations in the 3° structure elsewhere) or 3) differences in the capacity of these regions to bind to interacting proteins, all of which lead to the exposure or shielding of tryptic sites. These structural changes, regardless of their cause, may affect protein function *in vivo*.

The collagen VI microfibril is an ECM supramolecular assembly of heterotrimeric molecules comprised of alpha‐1, alpha‐2 and alpha‐3 chains, with the alpha‐3 chain being the largest (Cescon et al., 2015). Although previous histological analysis of photoaged skin showed no gross changes to collagen VI architecture (Watson et al., 2001), we recently demonstrated using peptide location fingerprinting that the alpha‐3 chain was structurally susceptible to physiological doses of UVR *in vitro* (Eckersley et al., 2020). As these ECM assemblies are long‐lived, here we show evidence for the first time that the alpha‐3 chain is susceptible to photoageing‐dependent modifications *in vivo* (Figure 3a). These regional peptide yield differences were located within N‐terminal vWF‐A domains and the collagenous region (Figure [Link], [Link]). Fibulin‐1 is an ECM‐associated protein in the dermis which influences cell activity. Although skin photoageing has been shown to affect the co‐localisation of fibulin‐2 and fibulin‐5 on elastic fibres (Kadoya et al., 2005), we demonstrate that the structure of fibulin‐1 may be affected due to the observation of N‐terminal differences in peptide yields (Figure 3b). One of these regions corresponds to a fibulin‐1 anaphylatoxin‐like domain (Figure [Link], [Link]) which plays a key role in self‐assembly (Tran et al., 1997). Biglycan is an ECM‐associated proteoglycan capable of transforming growth factor‐β (TGFβ) regulation (Kolb et al., 2001) whose presence is reduced in photoaged forearm dermis compared to intrinsically aged buttock (Lee et al., 2016). We show that dermal biglycan is also subjected to modification by photoageing which may impair functionality within regions (Figure 3c) corresponding to three of its leucine‐rich repeats (Figure [Link], [Link]). Although reductions in epidermal galectin‐7 expression (important in wound repair) was shown recently in intrinsically aged skin (Choi et al., 2018), we show that dermal galectin‐7 is additionally susceptible to photoageing (Figure 3d).

Epidermal K2 and K10 had evidence of photoageing‐dependent structural modifications evidenced by peptide yield differences (Figure 3e,f) within the coil domains (Figure [Link], [Link]). Both proteins are involved in keratinocyte differentiation and cornification (Zhu et al., 1999). Although the upregulation of several epidermal keratins has been shown in response to acute UVR exposure (Smith & Rees, 1994), to our knowledge, the impact of chronic UVR on K2 and K10 has not been demonstrated *in vivo*. Epidermal cells rely on desmosomal desmoplakin to mediate adhesion (Vasioukhin et al., 2001). Here, we show that photageing affects a region in desmoplakin (Figure 3g), corresponding to a plectin repeat on the C‐terminal side of the protein (Figure [Link], [Link]), which facilitates the adhesion of epithelial cells to the basement membrane (Meng et al., 1997). Finally, HSP70 s are molecular chaperones and crucial regulators of cell proteostasis (Fernández et al., 2017). Two 50 aa regions in HSP701A exhibited differences in peptide yield (Figure 3h), one of which lies within the nuclear‐binding region (Figure [Link], [Link]) responsible for hydrolysing ATP and regulating protein binding (Mayer & Bukau, 2005). In mice, HSP70 overexpression was shown to suppress UVR‐induced wrinkle formation highlighting its therapeutic potential (Matsuda et al., 2013). However, this is the first demonstration of photoageing‐dependent alterations to HSP70.

Peptide location fingerprinting successfully enabled the identification of local differences within protein regions revealing a panel of biomarker candidates with photoageing‐specific modifications. These may impact the functionality of skin, influencing multiple mechanisms of damage and, ultimately, skin homeostasis. For this reason, it is important to next consider these pathway‐level changes.

### 2.2 Pathway analysis of skin proteins with photoageing‐specific modifications to structure highlights ECM organisation, protein synthesis, keratinisation and hemidesmosome assembly as some of the networks most affected

**FIGURE 4:**
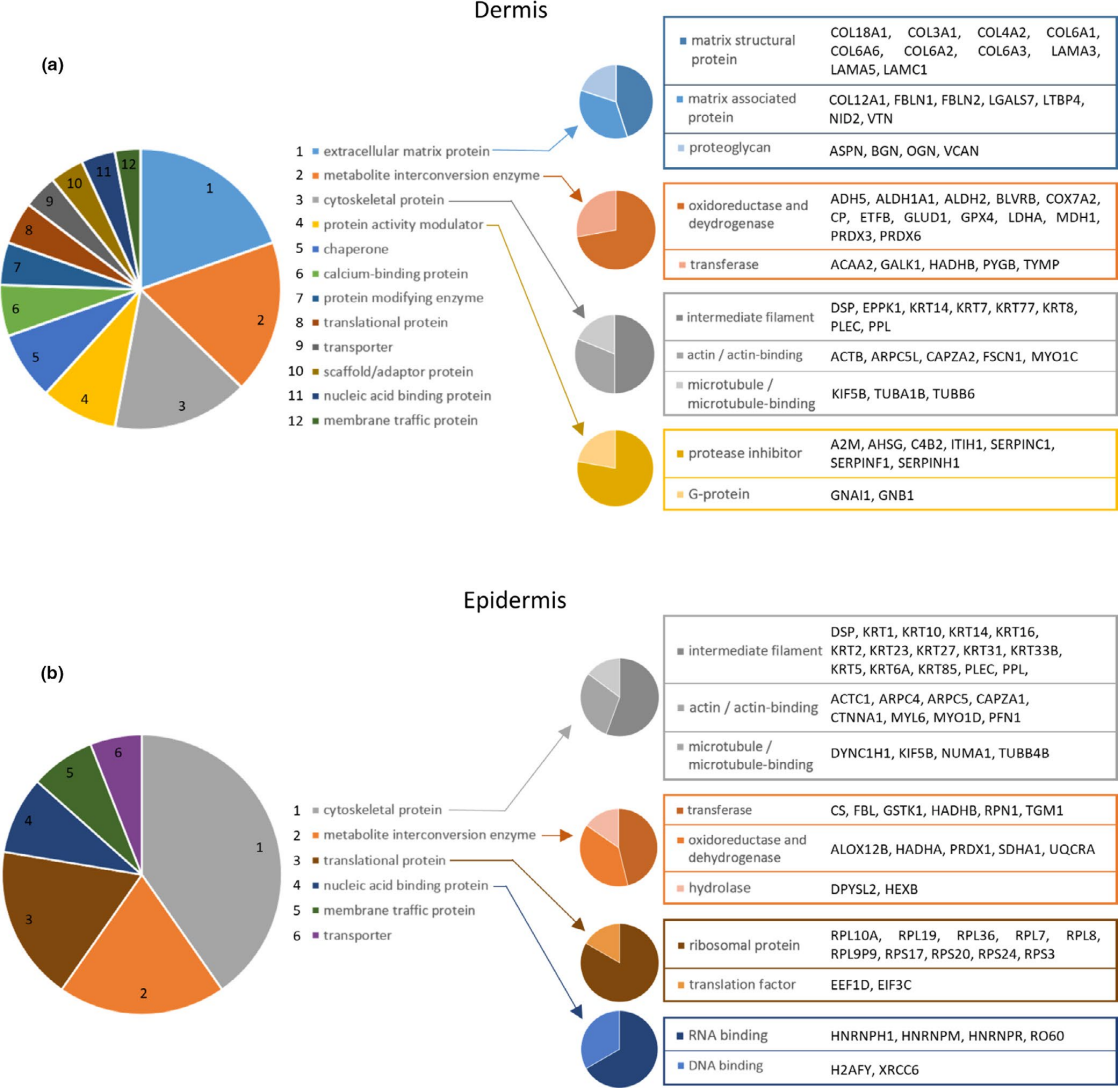
Classification of modification‐specific biomarker candidates into protein classes reveals ECM components, ROS‐ and protease‐modifying enzymes, cytoskeletal proteins and ribosomal proteins as the classes most affected by photoageing. Proteins with photoageing‐specific modifications were categorised into protein classes (PANTHER classification system; large multi‐coloured pie charts). The top four classes for dermis (a) were as follows: ECM proteins (blue slice), metabolite interconversion enzymes (orange slice), cytoskeletal proteins (grey slice) and protein activity modulators (yellow slice). Cytoskeletal proteins and metabolite interconversion enzymes were also in the top four classes for the epidermis (b) in addition to translational proteins (brown slice) and nucleic acid binding proteins (dark blue slice). Protein identities contained within sub‐categories are also listed

Classification analysis of proteins with significant differences in regional peptide yield highlighted ECM components as the most affected protein class in the dermis (Figure 4a). Common to both dermis and epidermis (Figure 4b), cytoskeletal proteins (top in epidermis) and metabolite interconversion enzymes were also among the top four classes most affected by photoageing. Protein activity modulators were confined to the dermis and translation and DNA‐/RNA‐binding proteins were classes exclusive to the epidermis.

To further investigate the potential consequences induced by the functional decline of these photoageing biomarker candidates in skin, enrichment (overrepresentation) analysis was performed using Reactome (Fabregat et al., 2018) to derive the pathways most likely to be significantly affected (Table 1; Full lists – Tables S7). The pathways most enriched in the dermis included ECM organisation (particularly proteoglycan activity and collagen assembly/degradation) and protein folding (Table 1). In the epidermis, affected pathways included keratinisation, eukaryotic translation and hemidesmosome assembly.

**TABLE 1:** Fifteen of the most significant Reactome pathways derived by enrichment analysis of photoageing biomarker candidates identified in skin by peptide location fingerprinting (selected from the top 25)

Dermis	Pathway name	Entities found	Entities FDR
1	Extracellular matrix organization	27	1.50E−13
2	Integrin cell surface interactions	10	1.46E−09
3	Neutrophil degranulation	26	1.46E−09
4	Assembly of collagen fibrils and other multimeric structures	12	5.61E−09
5	ECM proteoglycans	13	4.88E−08
6	Formation of tubulin folding intermediates by CCT/TriC	8	7.70E−08
7	Prefoldin mediated transfer of substrate to CCT/TriC	8	1.17E−07
8	Defective CFTR causes cystic fibrosis	4	5.98E−07
9	Immune System	56	5.98E−07
10	Chaperonin‐mediated protein folding	11	5.98E−07
11	Collagen degradation	9	5.98E−07
12	Collagen formation	13	1.10E−06
13	ABC transporter disorders	4	1.30E−06
14	Platelet degranulation	10	1.82E−06
15	Degradation of the extracellular matrix	13	2.20E−06

Epidermis	Pathway name	Entities found	Entities FDR
1	Formation of the cornified envelope	18	2.25E−10
2	Neutrophil degranulation	22	1.12E−08
3	Keratinization	18	1.39E−08
4	L13a‐mediated silencing of Ceruloplasmin expression	12	6.99E−08
5	Formation of a pool of free 40S subunits	11	2.72E−07
6	SRP‐dependent cotranslational protein targeting to membrane	11	5.09E−07
7	GTP hydrolysis and joining of the 60S ribosomal subunit	12	1.13E−06
8	Eukaryotic Translation	12	2.89E−06
9	Selenocysteine synthesis	10	2.91E−06
10	Peptide chain elongation	10	9.78E−06
11	Regulation of expression of SLITs and ROBOs	13	1.04E−05
12	Cap‐dependent Translation Initiation	12	2.94E−05
13	Selenoamino acid metabolism	11	2.94E−05
14	Nonsense‐Mediated Decay (NMD)	10	2.07E−04
15	Type I hemidesmosome assembly	5	4.39E−04

The dermis is comprised primarily of long‐lived structural ECM proteins (Sell & Monnier, 2010; Sivan et al., 2008) which may accumulate damage due to chronic UVR exposure, leading to changes in molecular abundance and function, ultimately contributing to the photoageing phenotype (Naylor et al., 2011). The majority of evidence for this is based on histological differences in elastic fibre (fibrillin‐1 and elastin) and fibrillar collagen architecture and abundance (Talwar et al., 1995; Watson et al., 1999). Although peptide location fingerprinting did identify significant differences in the protein regions within fibrillin‐1 and collagen I alpha chains (Table S5), the high intragroup variation of these regions between samples (Table S3) meant that it was difficult to attribute differences to either photoageing or individual variability. For proteins such as these, more samples may be required to identify photoageing‐specific regional changes. Regardless, peptide location fingerprinting revealed numerous, potentially novel ECM biomarker candidates meriting further study (Figure 4a), whose measured regions were not susceptible to individual variation.

Collagen formation, assembly and degradation were among the pathways enriched in dermis (Table 1) as a consequence of structural changes by photoageing within the alpha chains of collagens II, IV, VI, XII and XVIII, but also in procollagen C‐endopeptidase enhancer‐1 (PCOLCE) and cathepsin‐B. PCOLCE is crucial for driving the cleavage of type I procollagen by BMP1 during collagen formation (Zhu et al., 2019) whereas cathepsin‐B plays an integral role in ECM remodelling, particularly of collagen IV (Buck et al., 1992). In addition, the identified collagen alpha chains interact directly with a range of proteins and glycosaminoglycans (GAGs) (see network: Figure [Link], [Link]). These include heparin, whose interaction with collagen VI alpha‐3 can regulate the proliferation of mesenchymal cells (Atkinson et al., 1996), and lysyl oxidase, a cross‐linking enzyme crucial for ECM deposition (Xiao & Ge, 2012). The functional impairment of these collagen remodelling pathways may partly explain the loss of fibrillar collagens observed histologically in photoageing.

Proteoglycan activity was also among the pathways most affected in the dermis (Table 1). Significant differences in regional peptide yield were identified in versican, mimecan, biglycan and asporin with potential consequences for age‐related disease. For example, remodelling of biglycan (which can bind low‐density lipoprotein cholesterol within the arterial wall) is associated with the progression of atherosclerosis (Scuruchi et al., 2020). Biglycan can also modulate cytokine activity and ROS signalling through NADPH oxidases (NOXs), enabling the recruitment of macrophages and B cells and directly influencing the inflammasome pathway (Nastase et al., 2017). Versican has also been shown to colocalise with recruited neutrophils in the skin of UVB‐exposed mice (Kunisada et al., 2011). These proteoglycan biomarker candidates also interact with a variety of biomolecules (see network – Figure [Link], [Link]) including hyaluronic acid, whose increased presence and fragmentation in photoaged dermis (Tzellos et al., 2009) may be influenced by a disruption in its interactions with the biomarker candidates versican (Wight, 2017) and collagen VI alpha‐3 (Specks et al., 1992).

In addition to the remodelling of ECM components, chaperonin‐mediated protein folding was also identified as a potential pathway most affected by photoageing in the dermis. This is primarily as a result of several subunits of the T‐complex protein ring complex (TRiC) being identified as structurally altered between forearm and buttock, including chaperonin containing TCPs (CCTs)‐1, −2, −4, −5, −7 and −8. As the TRiC complex aids in the folding of approximately 5 – 10% of the entire proteome (Yam et al., 2008), the potential dysfunction of six out of its eight subunits may have wide implications on protein folding in the dermis. CCTs interact with a myriad of proteins (see network: Figure [Link], [Link]) including biomarker candidates identified in this study such as the TRiC complex substrates beta actin and tubulins B6 and A1B (Yam et al., 2008).

In the epidermal proteome, cornification and keratinisation were identified as pathways affected by photoageing (Table 1) primarily due to the identification of 12 different modified keratins (Figure 4b, grey pie chart). As well as cornification, these play a variety of roles within the epidermis from barrier function integrity (Salas et al., 2016) to keratinocyte differentiation and proliferation (Alam et al., 2011). The identification of a global change within the keratin superfamily is indicative of a wider functional decline of these crucial processes. In addition to keratins, involucrin and the cross‐linking keratinocyte transglutaminase (TGM), both of which are also instrumental to the process of cornification, and loricrin, which comprises 70% of the cornified envelope, were all identified as structurally modified. These also interact with the keratins within the wider cornification network (Figure [Link], [Link]).

Hemidesmosomes are adhesion complexes that anchor basal keratinocytes to the basement membrane at the dermal–epidermal junction. Their assembly was also among the pathways most enriched in epidermis, primarily due to the critical interactions between plectin and integrin α6β4 (ITA6 and ITB4) (see network: Figure [Link], [Link]). These are crucial for the assembly and stability of hemidesmosomes (De Pereda et al., 2009) with mutations in these components giving rise to epidermolysis bullosa, which is characterised by the detachment of the basal lamina from keratinocytes (Schumann et al., 2013). Therefore, photoaging‐dependent changes in the structure of the plectin‐integrin α6β4 complex may also have similar implications.

Epidermal peptide location fingerprinting revealed 10 ribosomal proteins affected by photoageing (Figure 4b, brown pie chart) resulting in the enrichment of several protein synthesis pathways (Table 1) including protein translation and peptide chain elongation which may be functionally perturbed. The deterioration of ribosome abundance and function during ageing has been shown previously in mice (Nakazawa et al., 1984). In our study, ribosomal biomarker candidates interact directly with a number of transcription factors (see network: Figure [Link], [Link]) including c‐Jun which is fundamental to cell proliferation, survival and cycle progression (Holmström et al., 2008) and COP9 signalosome subunit 5 (COPS5) which is crucial for the phosphorolytic activation of c‐Jun (Naumann et al., 1999). The induction of c‐Jun has been previously observed in UV‐irradiated skin (Fisher et al., 1998), indicating a possible role in cell repair mechanisms.

### 2.3 Peptide location fingerprinting reveals biomarker candidates of photoageing which are not identified by conventional relative quantification of protein abundance

In addition to regional protease susceptibility using peptide location fingerprinting, protein abundance was relatively quantified within the same LC‐MS/MS data sets by MS1 peak area ion intensity. This was performed in order to identify biomarker candidates unique to both methodological approaches. A total of 635 dermal and 926 epidermal proteins were significantly different in relative abundance (Figure [Link], [Link]) between forearm and buttock according to peak area intensity (PCAs indicated clear data separations, Figure [Link], [Link]; protein list ‐ dermis: Table S8, epidermis: Table S9). As seen for peptide location fingerprinting, classification analysis also identified cytoskeletal proteins, metabolite interconversion enzymes and translational proteins as the top three classes whose relative abundance was affected in forearm skin (Figure [Link], [Link]).

Two new potential photoageing biomarker candidates, TIMP3 (metalloprotease inhibitor 3) in dermis and RPL36 (ribosomal protein L36) in epidermis which were identified as significantly different in relative abundance between forearm and buttock skin by MS1 intensity, were experimentally validated with Western blotting (Figure [Link], [Link]). Western blotting on the same protein extracts corroborated a significant increase in TIMP3, and decrease in RPL36, in forearm compared to buttock. All four TIMPs have been shown to inhibit all 26 MMPs (Murphy, 2011). One proposed mechanism of photoageing is that chronic sun exposure to fibroblasts and keratinocytes can lead to enhanced MMP activity in skin (Sárdy, 2009). The elevated presence of TIMP3 in forearm dermis is perhaps indicative of a heightened proteolytic environment and an attempt to mitigate UVR‐induced remodelling.

**FIGURE 5:**
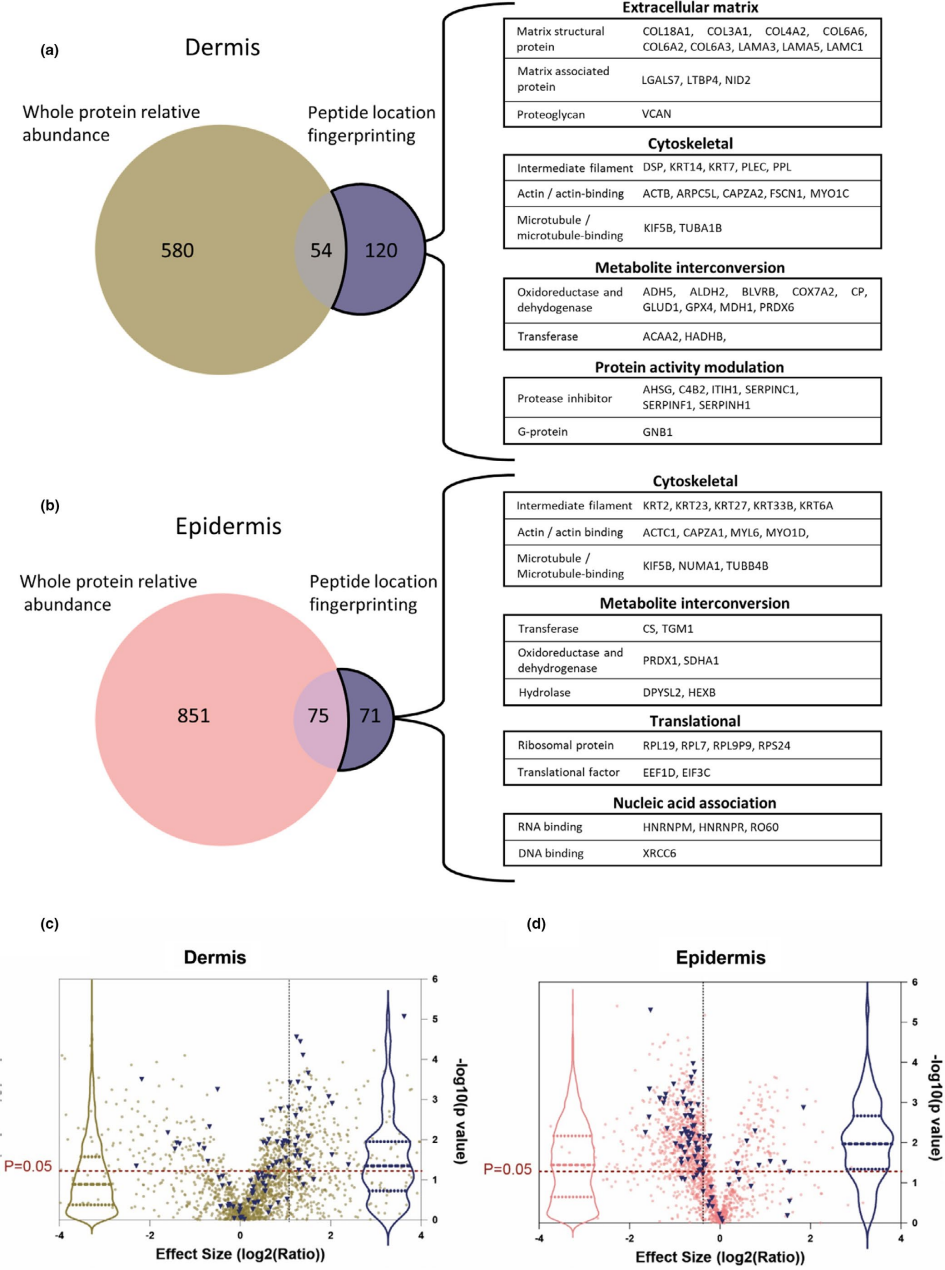
Peptide location fingerprinting identifies unique modification‐specific skin biomarker candidates of photoageing which were not identified by whole protein relative quantification. Venn diagrams compare the number photoageing‐modified protein biomarker candidates identified by peptide location fingerprinting with the number identified with significant differences in whole protein relative abundance by quantification with MS1 intensity. Several proteins were uniquely identified by peptide location fingerprinting in both dermis (a) and epidermis (b) including multiple ECM proteins and protein activity modulators for dermis, translation and nucleic acid‐associated proteins for epidermis and cytoskeletal proteins and metabolite interconversion enzymes for both sub‐tissues. Protein identities with significant modifications in structure correlated less strongly with significant differences in protein abundance in the dermis (c) than in the epidermis (d). Proteins with photoageing‐specific structural modifications were identified and labelled blue on relative protein abundance volcano plots from Figure [Link], [Link]. Violin plots (dashed lines = median and IQR) of the labelled points (blue) were superimposed to show correlation between proteins with modification‐associated differences and protein abundance differences (violin plots of all data points also shown in pink and gold). For the epidermis, a large proportion of proteins which had structural modifications (blue points and blue violin plot) are positioned above the *p* = 0.05 line, indicating that these were also significantly different in relative abundance between photoaged forearm and intrinsically aged buttock. This suggests that for epidermis, structure‐related differences appear to correlate strongly with differences in abundance (median *p*‐value = 0.01). For the dermis, however, a smaller proportion of proteins identified with modification‐associated differences are positioned above the *p* = 0.05 line suggesting that these differences appear to correlate less with abundance (median *p*‐value = 0.04).

Peptide location fingerprinting uniquely identified 120 protein biomarker candidates in the dermis (Figure 5a; Table S10) and 71 in the epidermis (Figure 5b; Table S11), which were modified as a consequence of photoageing but did not differ significantly in relative abundance. This demonstrates that changes in protein abundance do not necessarily correlate with changes related to protein modification, particularly in ECM‐rich tissues. Of these unique candidates, thirteen were ECM‐associated proteins including four collagens, two elastic fibre‐binding proteins (LTBP4 and nidogen‐2), three laminin chains and the proteoglycan versican. Protein classification analysis ranked ECM proteins as the most affected class in the dermis for structural modifications (Figure 4), but not for changes in relative abundance (Figure [Link], [Link]). In epidermis, peptide location fingerprinting also successfully identified photoageing modifications in numerous keratins, serpins and ribosomal proteins which did not significantly differ in abundance.

To test whether structure‐associated differences in proteins correlate with differences in relative abundance, p‐values of relative abundance differences (measured by MS1 intensity) of proteins identified with modifications (measured by peptide location fingerprinting) were compared between epidermis and dermis (Figure 5c,d). The proportion of proteins with both significant modifications and also significant differences in relative abundance was higher in epidermis than in the dermis. This observation may be reflective of the higher protein turnover in the epidermis compared to the dermis, further highlighting the potential of peptide location fingerprinting in elucidating biomarker candidates of chronic disease, particularly in ECM‐rich connective tissue.

### 2.4 The MPLF webtool can be readily applied to existing LC‐MS/MS data sets to reveal previously undiscovered ageing biomarker candidates

To showcase that peptide location fingerprinting can be used as a surveying tool to reveal novel ageing biomarker candidates by post hoc analysis of published data sets, the MPLF webtool was used to analyse an independent, publicly available LC‐MS/MS human tendon data (downloaded from the PRIDE repository; PXD006466). Hakimi et al., (2017) recently published a quantitative proteomic comparison between aged (torn) and young human supraspinatus tendon. They revealed significant reductions in the multiple pericellular ECM components such as collagens I and VI and elastic fibre proteins fibrillin‐1, microfibril‐associated protein 5 (MFAP5), LTBP2 and fibulin‐1.

**FIGURE 6:**
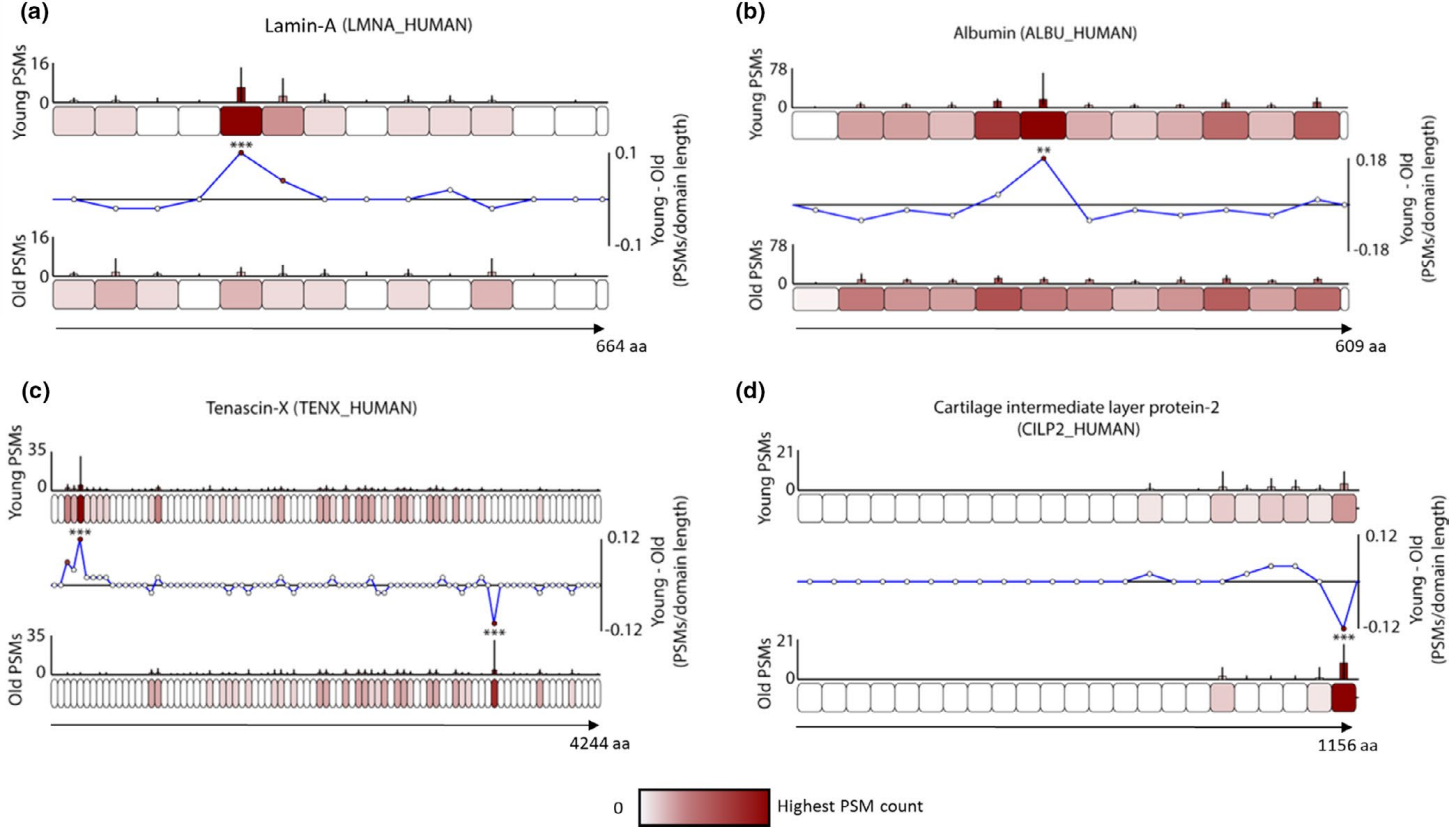
Manchester Peptide Location Fingerprinter webtool analysis of publicly available ageing human tendon data demonstrates that peptide location fingerprinting can be applied post hoc to downloaded LC‐MS/MS data sets to reveal unique structural modification biomarker candidates. Differences in peptide yield along the protein structure of four of these biomarkers identified are shown (a–d). Proteins are segmented into 50 aa‐sized step regions with average peptide counts (*N* = 9) heat mapped to each step and compared between aged and young groups (bar graphs = average PSMs, error bars = SD). Average peptide counts corresponding to each protein step in the aged group were subtracted from the counts of their corresponding protein step in the young group and divided by the aa sequence length of that step to reveal regional differences in peptide yield (line graphs) with statistically significances between groups shown (** ≤0.01, *** ≤0.001; Bonferroni‐corrected unpaired ANOVA). Both lamin‐A (a) and albumin (b) contained one N‐terminal sided region with significantly higher peptide counts in young compared to aged samples. Tenascin‐X contained one N‐terminal sided region which yielded significantly higher peptides in young compared to aged and another on C‐terminal side which yielded significantly lower peptide counts (c). A C‐terminal region of CILP2 also had significantly lower counts in young compared to aged (d)

Peptide location fingerprinting on this data set identified 54 regions with significant differences in peptide yield between aged and young male tendon corresponding to 31 proteins with possible modifications to structure (Table S12) Of these, lamin‐A, albumin, tenascin‐X and CILP2 are displayed (Figure 6a–d).

In common with those identified by Hakimi et al., (2017) by relative quantification, peptide location fingerprinting identified collagen I alpha‐1, collagen VI alpha 2, fibrillin‐1, LTBP2, complement and CILP2 as modification‐specific candidates of tendon ageing. However, a number of newly identified biomarker candidates exclusive only to protein structural modifications were also identified. These were collagen V alpha‐1, the proteoglycans versican, mimecan and asporin, albumin, the nuclear intermediate filament protein lamin A, and the cytokine TGFβ.

The application of peptide location fingerprinting to previously published human tendon data highlights the wider impact of this approach as a novel surveying tool capable of enhancing biomarker discovery in combination with conventional relative quantification methods.

## 3 CONCLUSION

Peptide location fingerprinting of LC‐MS/MS data sets generated from photoaged forearm and intrinsically aged buttock skin enabled the identification of proteins with structure‐associated modifications specific to chronic sun exposure. As well as a biomarker discovery tool, we demonstrated that this approach can be used to investigate local molecular differences in peptide yield within structural regions of proteins in complex, whole tissue lysates. Crucially, peptide location fingerprinting revealed novel biomarker candidates of photoageing which remain undetectable by conventional relative quantification, in particularly for long‐lived ECM proteins.

Through the use of the MPLF webtool, peptide location fingerprinting can be applied to any LC‐MS/MS data set suitable for data‐dependent acquisition. As such, a combinational approach of both peptide location fingerprinting and relative protein quantification enables a more complete assessment of tissue proteostasis as a consequence of chronic disease and creates a powerful tool for biomarker discovery.

## 4 MATERIALS AND METHODS

For methods on tissue histology, staining and imaging, Western blotting and label‐free quantification of protein using Progenesis QI, please see Appendix S1.

### 4.1 Human tissue and materials

Chemicals were sourced from Sigma‐Aldrich (Poole, UK) unless otherwise stated. Human skin was collected from aged donors (*N* = 7, mean age = 70 years, range = 62–79 years, 3 males; Fitzpatrick skin phototype I‐III) with informed and written consent following approval from The University of Manchester Research Ethics Committee (ref: UREC 15464). Samples were bisected and snap‐frozen for LC‐MS/MS and histology.

### 4.2 Sample preparation for mass spectrometry

Employing any LC‐MS/MS analysis on skin dermis is challenging as networking ECM structures are highly insoluble, tightly bonded, highly modified and covalently crosslinked. An optimised protocol was devised which maximised peptide yield in dermis and epidermis. As peptide location fingerprinting measures the peptide yield within regions across protein structures, it is heavily reliant on coverage. Previously, we have used porcine elastase to maximise protein coverage from purified fibrillin and collagen VI microfibrils in proof of concept studies using peptide location fingerprinting (Eckersley et al., 2018, 2020). The relative non‐specificity of elastase compared to trypsin (which cleaves exclusively at the C‐term end of Arg and Lys) was appropriate due to the relative simplicity of these proteomes. However, the high complexity and protein diversity of tissue proteomes meant that elastase would generate a proportion of peptides with identification confidences too low for use in this study. Here, trypsin successfully yielded an adequate number of high confidence peptides (FDR = 0.6%) to provide suitable coverage across hundreds of proteins. Nevertheless, the use of alternative or additional enzymes (e.g. elastase, chymotrypsin and Lys‐C) or sample enrichment to maximise peptide coverage across proteins is an important consideration for future studies.

Skin samples were incubated in 20 nM EDTA in PBS for 2 hrs at 37°C to ensure dermal–epidermal separation. Biopsies were washed in PBS and the epidermis separated. Dermis was minced and incubated in 8 M urea buffer (+ 25 mM ammonium bicarbonate +25 mM dithiothretol [DTT]) and homogenised using a bullet blender (Next Advance, NY, USA). The epidermis was homogenised in 8 M urea buffer with an ultrasonicator (Covaris, Brighton, UK) with 175 W peak power for 8 min. All samples were centrifuged and supernatants diluted to 2 M urea with AB buffer (25 mM ammonium bicarbonate and 1.3 mM calcium chloride) prior to digestion with trypsin SMART Digest Beads (Thermo Fisher, MA, USA), overnight at 37°C. Peptide samples were reduced in 10 mM DTT for 10 min at 60°C, alkylated in 30 mM iodoacetamide for 30 min and acidified in 2% (v/v) trifluoroacetic acid. Biphasic extraction was performed via agitation with ethyl acetate. Peptides were then desalted using OLIGO R3 ReversedPhase Resin (Thermo), vacuum dried and re‐suspended in 5% acetonitrile (ACN) prior to LC‐MS/MS analysis (~10 μg injections).

### 4.3 Mass spectrometry

Peptide mixtures were analysed by LC‐MS/MS using an UltiMate^®^ 3000 Rapid Separation Liquid Chromatography system (Dionex, CA, USA) coupled to a Q Exactive HF mass spectrometer (Thermo). Peptide mixtures were separated using a multistep gradient from 95% A (0.1% formic acid [FA] in water) and 5% B (0.1% FA in ACN) to 7% B at 1 min, 18% B at 58 min, 27% B at 72 min and 60% B at 74 min at 300 nL/min, using a 250 mm × 75 μm i.d. 1.7 µM CSH C18, analytical column (Waters, Hertfordshire, UK). Peptides were selected for fragmentation automatically by data‐dependant analysis.

### 4.4 Peptide list generation for peptide location fingerprinting

Raw MS spectrum files were converted to Mascot MGF files using ExtractMSn (Thermo) under default parameters and submitted to the Mascot server. The Mascot search engine then correlated the peak spectra within each to the UniProt human database (2018; Swiss‐Prot and TreEMBL). Mascot MS/MS ion searches were performed with the following parameters: database—Swissprot_TreEMBL_2018_01; species—*Homo sapiens*; enzyme—trypsin; peptide charge—2+ and 3+; max missed cleavages—2; fixed modifications—carbamidomethyl (mass: 57.02 Da; aa: C); variable modification—oxidation (mass: 15.99 Da; aa: M); peptide tolerance—10 ppm; fragment tolerance—0.02 Da; and instrument—ESI‐TRAP.

Mascot search results were exported as DAT files. PSMs were generated using the Scaffold 4 (Proteome Software; OR, USA) and peptide/protein identifications generated automatically using LFDR scoring. Data were filtered to report peptides exclusive to their matched proteins. Peptide probability was thresholded to 95% minimum giving a low peptide false discovery rate (FDR) of 0.5% for epidermis samples and 0.6% for dermis. Peptide FDR was calculated by Scaffold 4 using peptide probabilities assigned by the Trans‐Proteomic Pipeline (WA, USA) using the PeptideProphet algorithm. Peptide lists used for peptide location fingerprinting were exported from Scaffold 4 (Tables S1 and S2) within CSV files which were imported into the webtool.

### 4.5 Peptide location fingerprinting using the Manchester peptide location fingerprinting webtool

The MPLF webtool is a publicly available LC‐MS/MS analysis tool developed in‐house, integrated within our published database: the Manchester Proteome (Hibbert et al., 2018), which performs high‐throughput peptide location fingerprinting analysis of LC‐MS/MS data:

https://www.manchesterproteome.manchester.ac.uk/#/MPLF. A user manual is also available on the webpage. Calculations are performed by MPLF on the server side with a code written in Python version 3.8. The back end is powered by Python Django 3.8 framework and by mySQL 8, and the front end operates REACTjs with Redux state management.

The concept of peptide location fingerprinting by the MPLF webtool was designed using both LC‐MS/MS spectral counting data and MS1 peak area ion intensity; however, for this study, we used spectral count instead of MS1 intensity. A cornified, ECM‐rich and aged organ such as skin gives very complex MS1 intensities resulting in multiple signals from the same peptide. This is because long‐lived proteins contain a highly complicated array of modifications (e.g. the high abundance of collagen proline modifications) which lead to changes in mass and differences in shape resulting in the same peptide eluting at multiple retention times on the liquid chromatography column, hence introducing noise. At the MS1 level, this is challenging to filter out from these data sets and interferes with the meaning of the results. Spectral count deals with multiple instances of peptides very well since, rather than summing a large number of intensity values which vary heavily for the same peptide, we are instead summing individual counts. As a result, spectral counting smoothens the data and is therefore better suited to measuring the gross changes in relative quantification necessary for long‐lived cornified and ECM proteins in this study. As such, here, we have built our pipelines for photoaged, cornified‐heavy tissues with spectral counting in mind as it gives us the best answer to our biological question.

We have added MS1 intensity analysis as a functionality of the MPLF webtool but we urge caution due to the complexity of peptide identification at the tissue level. Therefore, we highly recommend the use of spectral count, or that the data are heavily filtered, if using complex ECM‐containing tissue data sets. We hope to continue developing the MPLF webtool and adjust in future to better incorporate MS1 level analysis and additional organism proteomes.

Peptide list CSVs (Tables S1 and S2) were imported into MPLF and PSMs were mapped to and automatically summed per respective regional 50 aa step within a protein, normalised based on individual protein total spectrum count, averaged per group (forearm or buttock; *N *= 7) and subsequently heat mapped onto representative schematics of each protein. Peptide sequences which spanned two adjacent regions were counted within both. Average PSM counts corresponding to 50 aa number step sizes within protein structures of one group (buttock) were then subtracted from the counts of their corresponding step sizes in the other group (forearm) and divided by each step's aa length to show regional differences in peptide yield. PSM counts corresponding to each aa step size were statistically compared between buttock and forearm groups using Bonferroni‐corrected, repeated measures paired ANOVA.

### 4.6 Pathway enrichment and network analysis

Pathway enrichment analysis was performed using Reactome (Fabregat et al., 2018). The database was queried using separate lists of epidermal and dermal biomarker candidate UniProt IDs (Consortium, 2016) and expanded to including IntAct database‐derived interactors. An overrepresentation analysis, corrected for FDR using the Benjamani–Hochberg method, was then performed by Reactome to determine the pathways enriched by the submitted lists. Only pathways of FDR ≤0.001% were considered significant (Table 1, Table S7). Protein entities pertinent to these pathways were subsequently used to build pathway‐ or protein class‐specific, experimentally identified interaction networks. MatrixDB (Launay et al., 2015) was used to derive ECM proteins interactions and IntAct (Orchard et al., 2014) for all others. These were then downloaded and visualised using Cytoscape 3.4.0.

## CONFLICT OF INTEREST

The authors have no conflicts of interest. Walgreens Boots Alliance approved the submission but exerted no editorial control.

## AUTHOR CONTRIBUTIONS

MO and AE contributed equally to this work. MO and AE conceived, designed and performed all proteomic experiments, analysed all data and prepared the figures. MO programmed and developed the MPLF webtool. AE conceptualised the application of peptide location fingerprinting, wrote the paper, performed the pathway analyses and Western blot experiments. MJS contributed to conception, design, data interpretation, preparation of figures and to the writing. KTM was responsible for tissue procurement and performed all histology, staining and microscopy. VM and JS contributed to design of MS sample preparation. SW, ROC and DK provided technical support for all MS performed and to the interpretation of data. CEMG and REBW and contributed to the interpretation of results. JS contributed to the conceptualisation of intragroup variation testing. All authors contributed to editing of the paper.

## Supporting information

Fig S1‐S15

Tab S1

Tab S2

Tab S3

Tab S4

Tab S5

Tab S6

Tab S7

Tab S8

Tab S9

Tab S10

Tab S11

Tab S12

App S1

App S2

## Data Availability

MPLF webtool access: https://www.manchesterproteome.manchester.ac.uk/#/MPLF. MS data have been deposited to the PRIDE repository. Dataset identifier ‐ PXD021194 and 10.6019/PXD021194. Skin proteins identified by LC‐MS/MS were used to expand the existing Manchester Proteome database ‐ https://www.manchesterproteome.manchester.ac.uk/#/Proteome.
