# Supplementary material for: Peptide location fingerprinting reveals modification‐associated biomarker candidates of ageing in human tissue proteomes
Source: Aging Cell. 2021 Apr 8;20(5):e13355. doi: 10.1111/acel.13355 (PMC8135079; doi:10.1111/acel.13355)

Table S1. Full list of LC-MS/MS-identified exclusive peptides in dermis samples used for structural mapping and fingerprinting.

Table S2. Full list of LC-MS/MS-identified exclusive peptides in epidermis samples used for structural mapping and fingerprinting.

Table S3: Intragroup variation testing of dermal protein regions

Table S4: Intragroup variation testing of epidermal protein regions

Table S5. Full list of peptide location fingerprinted dermal protein regions with significantly different peptide counts between forearm and buttock

Table S6. Full list of peptide location fingerprinted epidermal protein regions with significantly different peptide counts between forearm and buttock

Table S7. Full list of enriched Reactome pathways ranked by significance for epidermis and dermis photoageing biomarker candidates.

Table S8. Full list of relatively quantified dermal proteins by peak ion intensity ranked by significance.

Table S9. Full list of relatively quantified epidermal proteins by peak ion intensity ranked by significance.

Table S10: Dermal protein biomarkers identified exclusively by peptide location fingerprinting, relative protein quantification or by both methodologies.

Table S11: Epidermal protein biomarkers identified exclusively by peptide location fingerprinting, relative protein quantification or by both methodologies.

Table S12: Full list of peptide fingerprinted tendon protein regions with significantly different peptide counts between old and buttock

Figure S1. Representative histology images of biopsies to visually showcase photoageing phenotype.

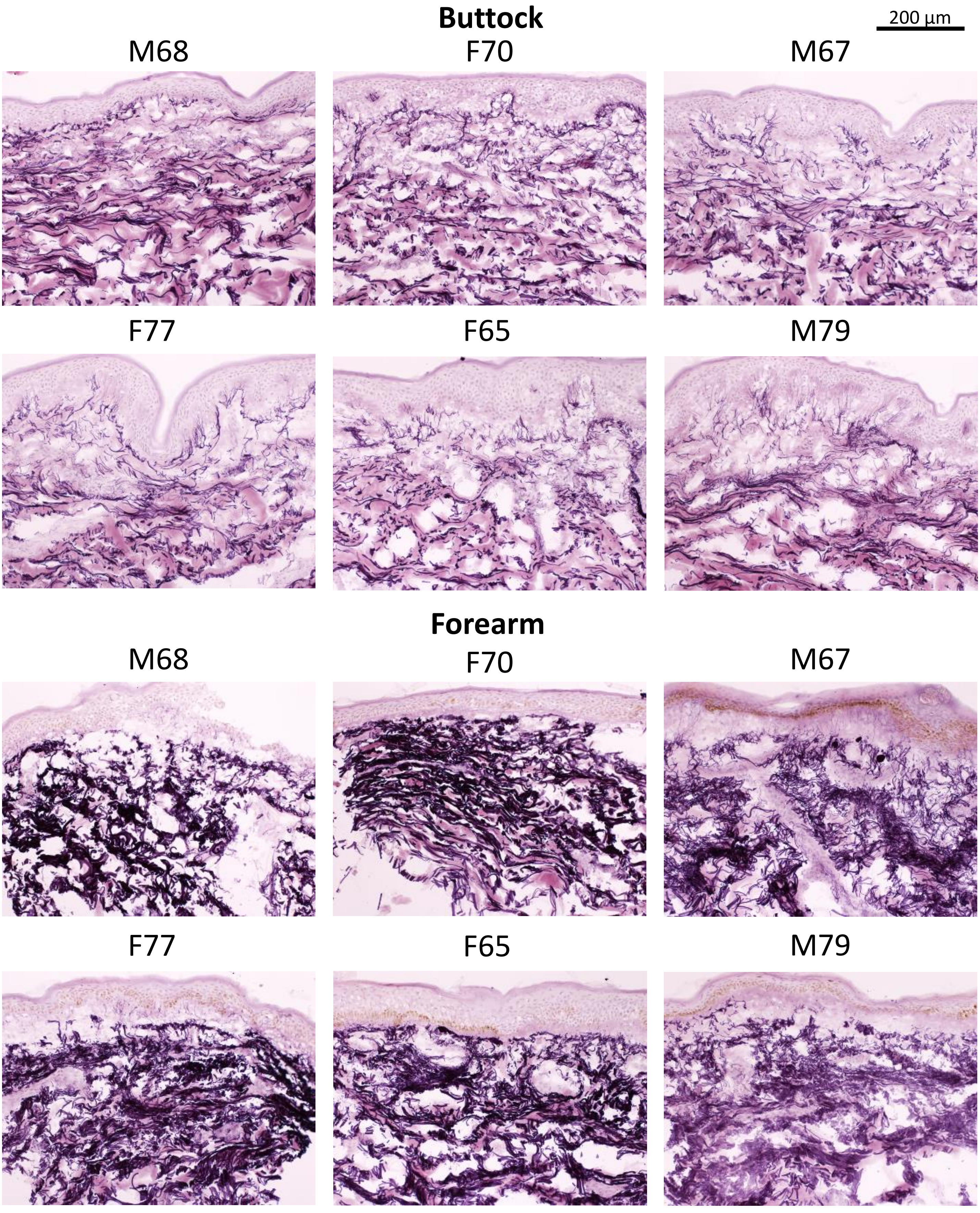

Figure S2. Skin samples from photoexposed forearm were severely photoaged compared to photoprotected buttock.

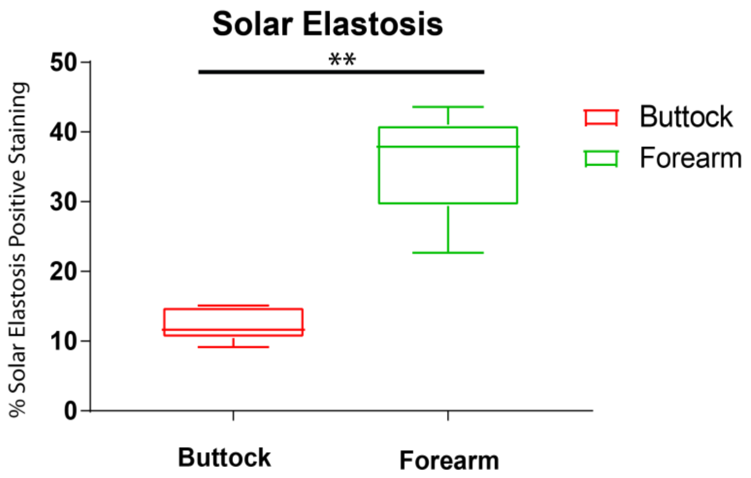

Figure S3. Principal component analysis (PCA) with partitioning around medoids clustering (red and green ellipses) of spectral count data used for peptide location fingerprinting.

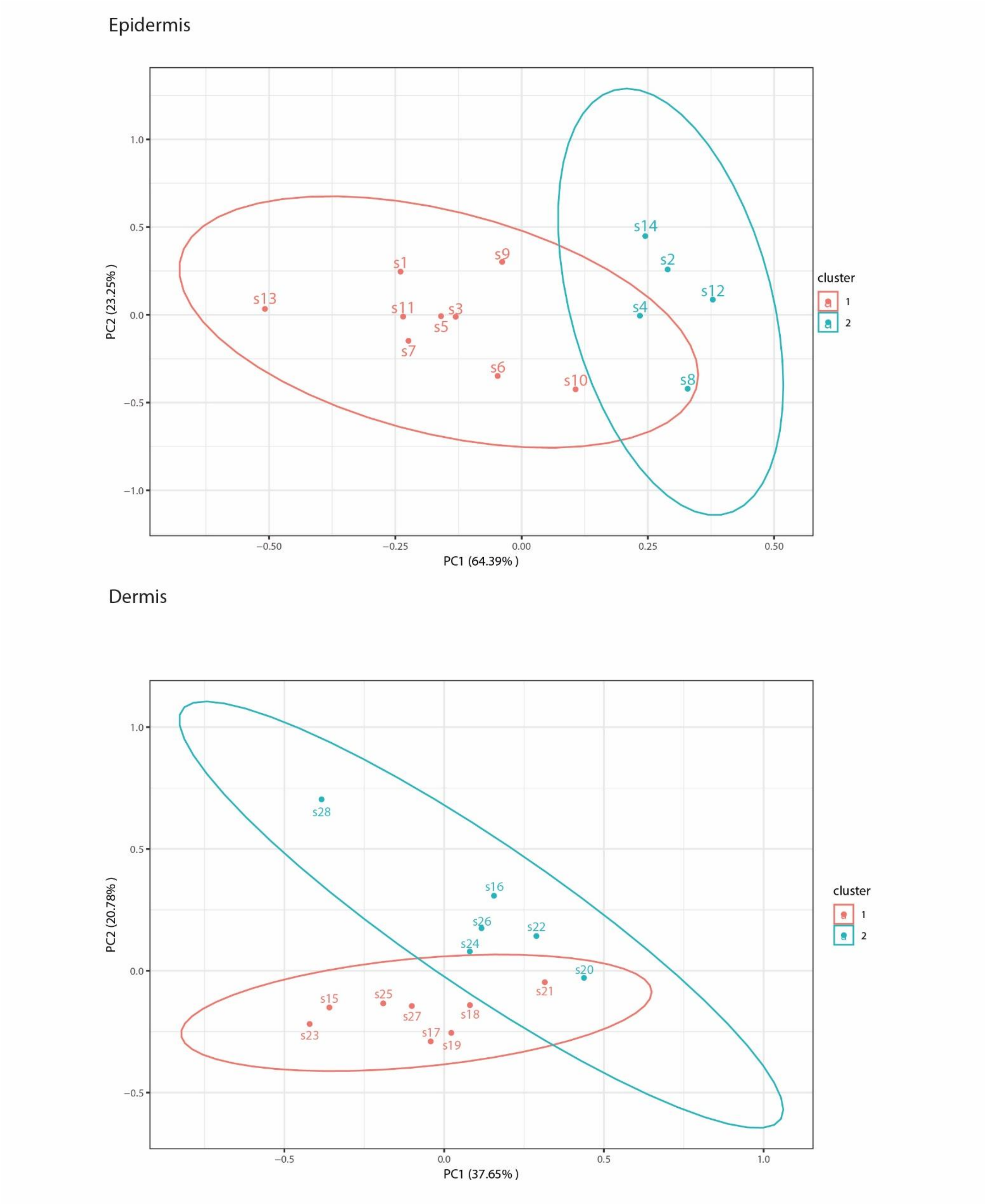

Figure S4. Exemplary biomarkers exhibiting photoageing-specific structural modifications - mean + standard deviations

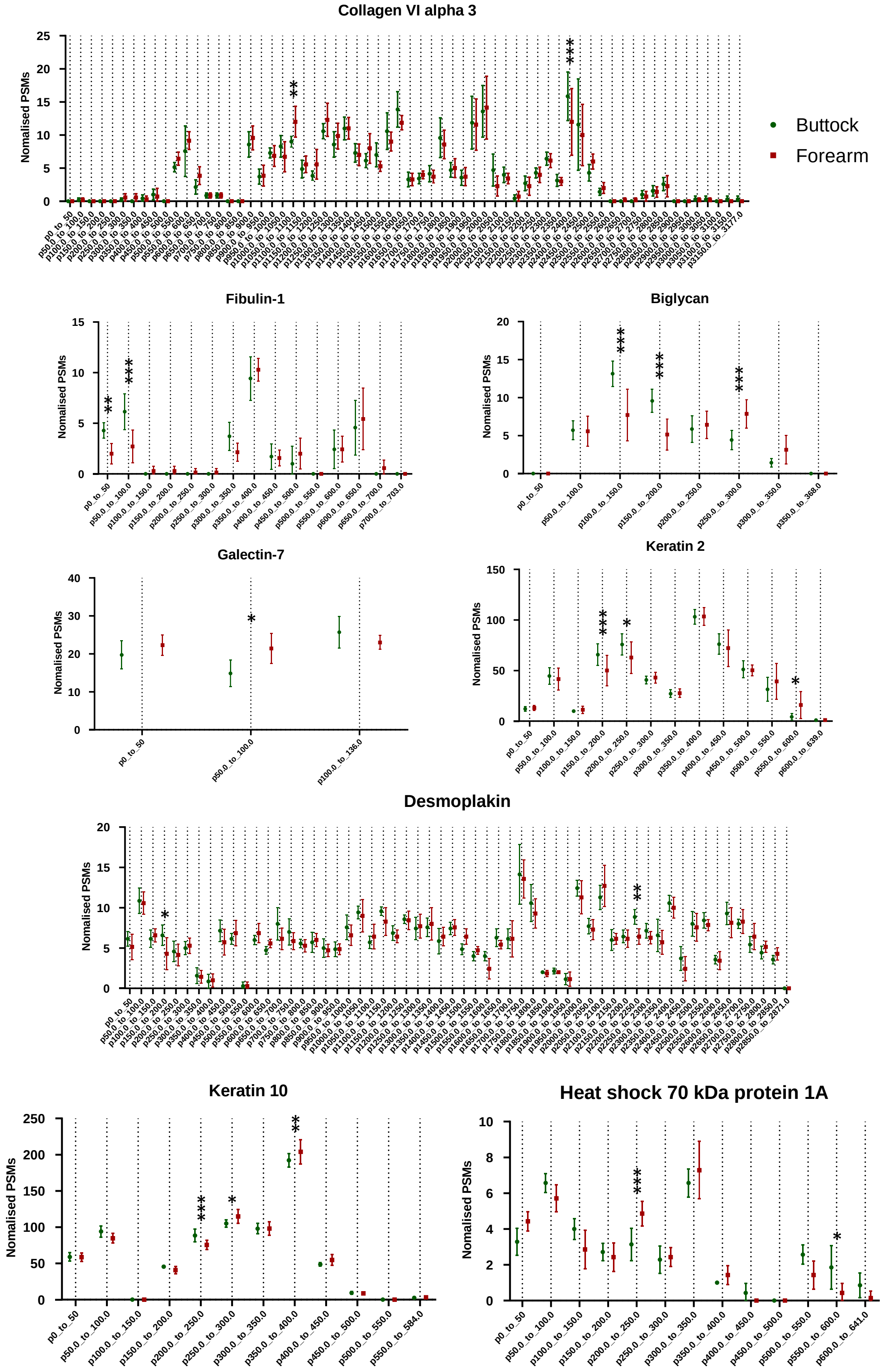

Figure S5. Peptide location fingerprinting of conserved domains, regions or repeats within exemplary biomarkers (Fig. 3).

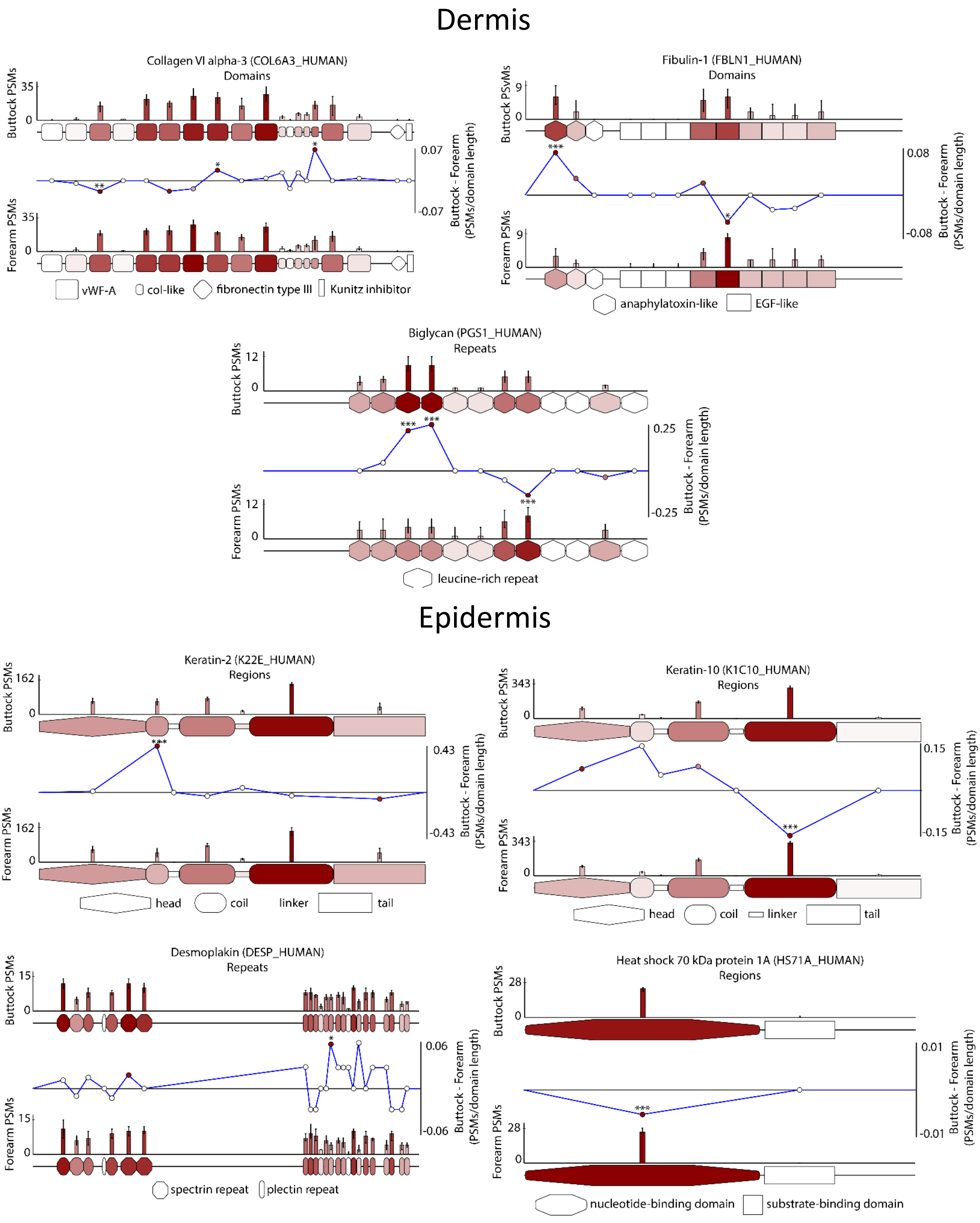

Figure S6. Collagens and their direct experimental interactors, curated from Matrix DB.

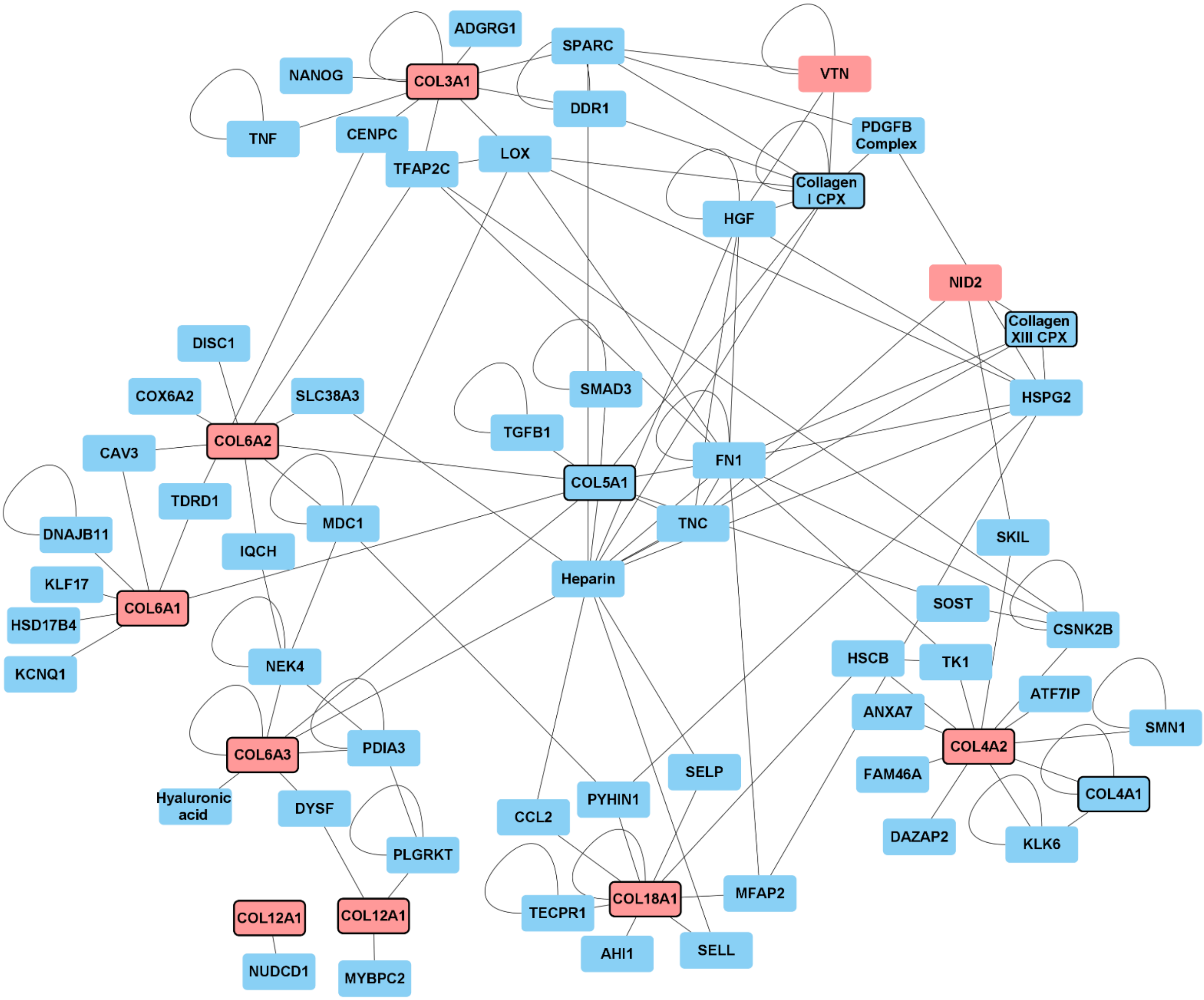

Figure S7. Proteoglycans, GAGs and their direct experimental interactors, curated from Matrix DB.

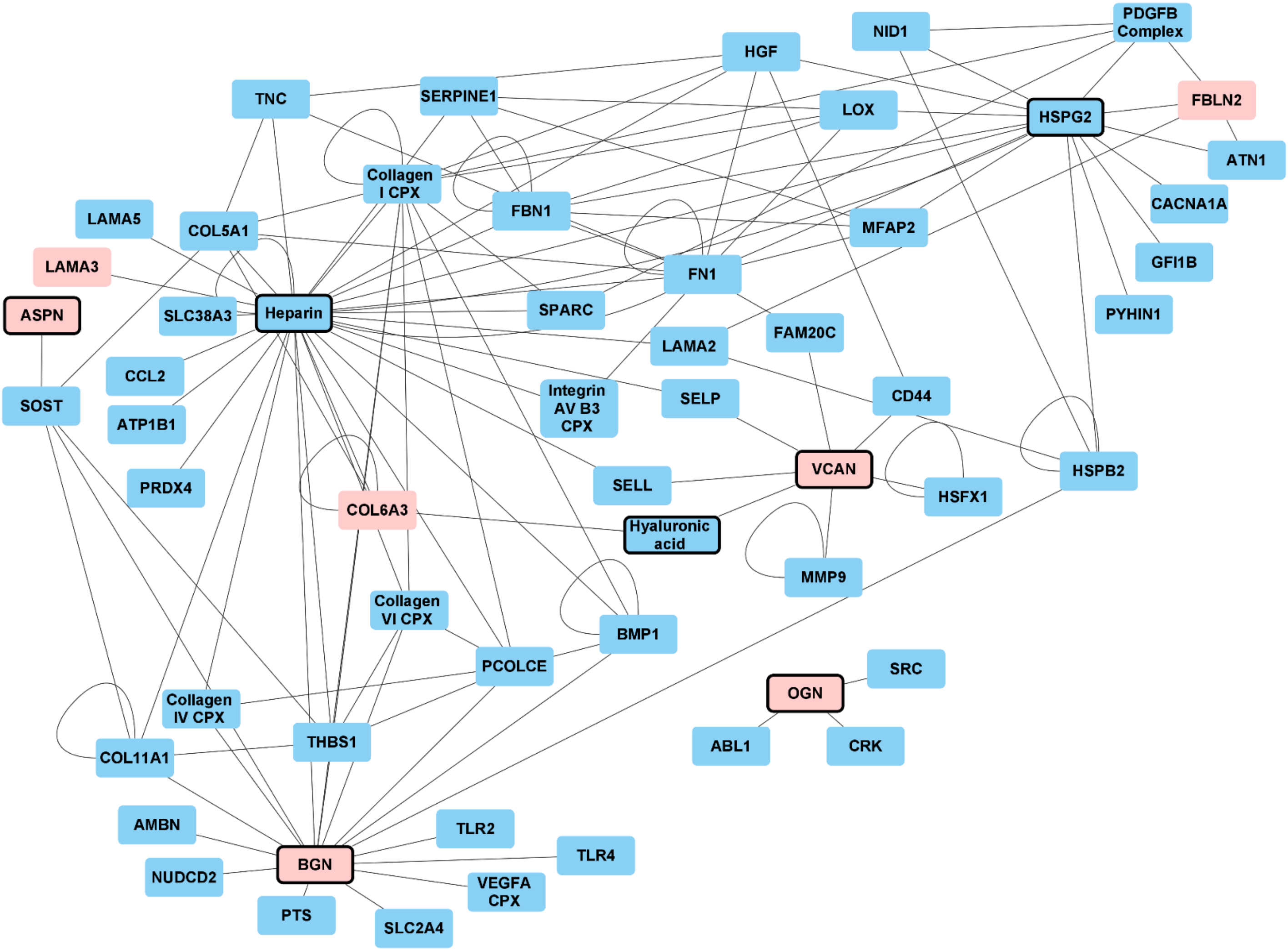

Figure S8. TRiC complex subunits (CCTs) and their direct experimental interactors, curated from the IntAct database.

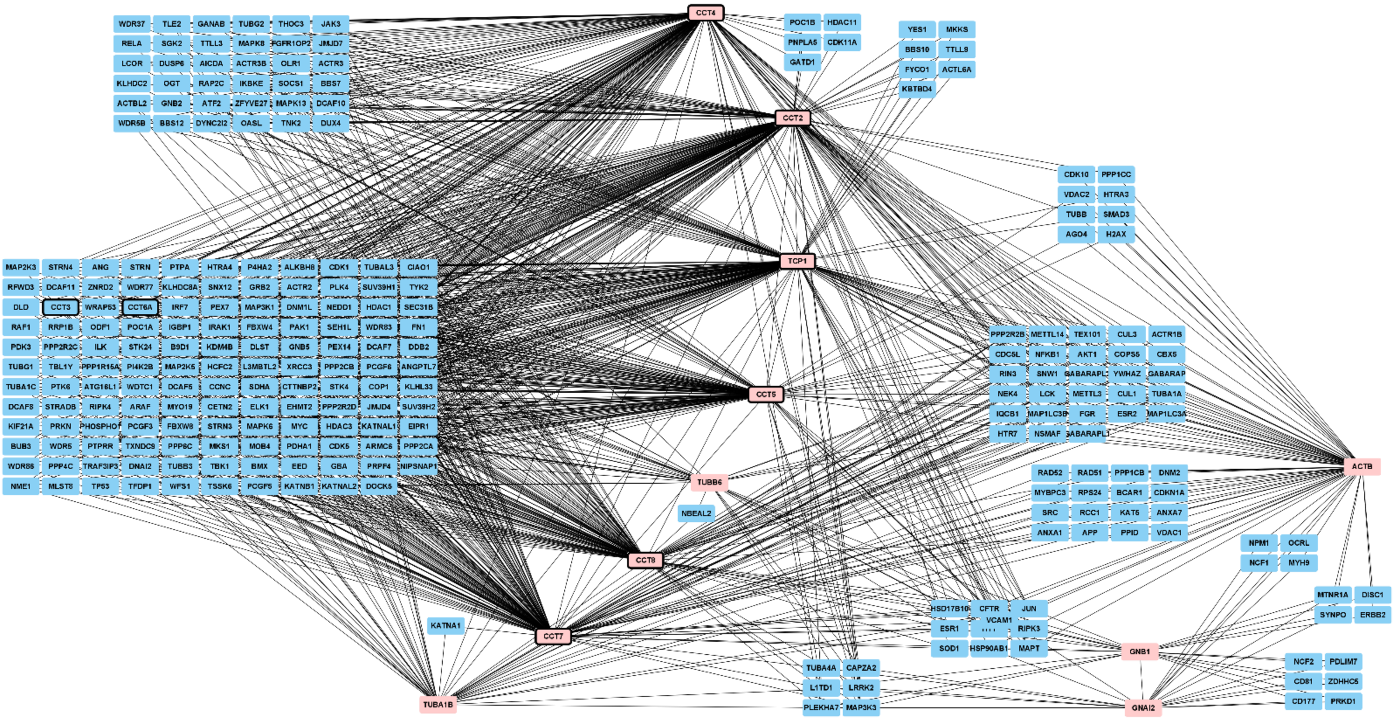

Figure S9. Cornification proteins and their direct interactors, experimentally derived using IntAct.

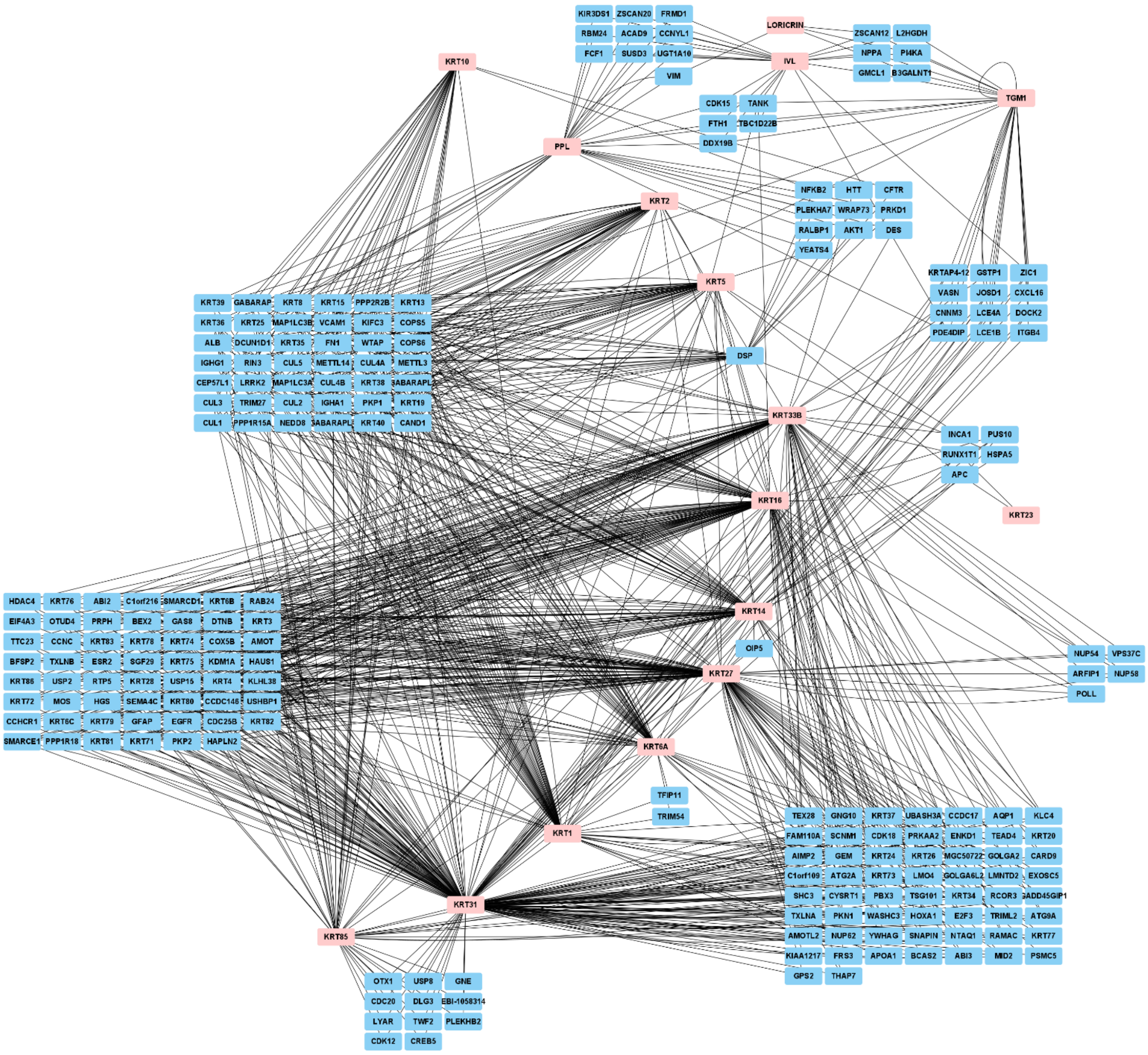

Figure S10. Hemidesmosome assembly proteins and their direct interactors, experimentally derived using IntAct.

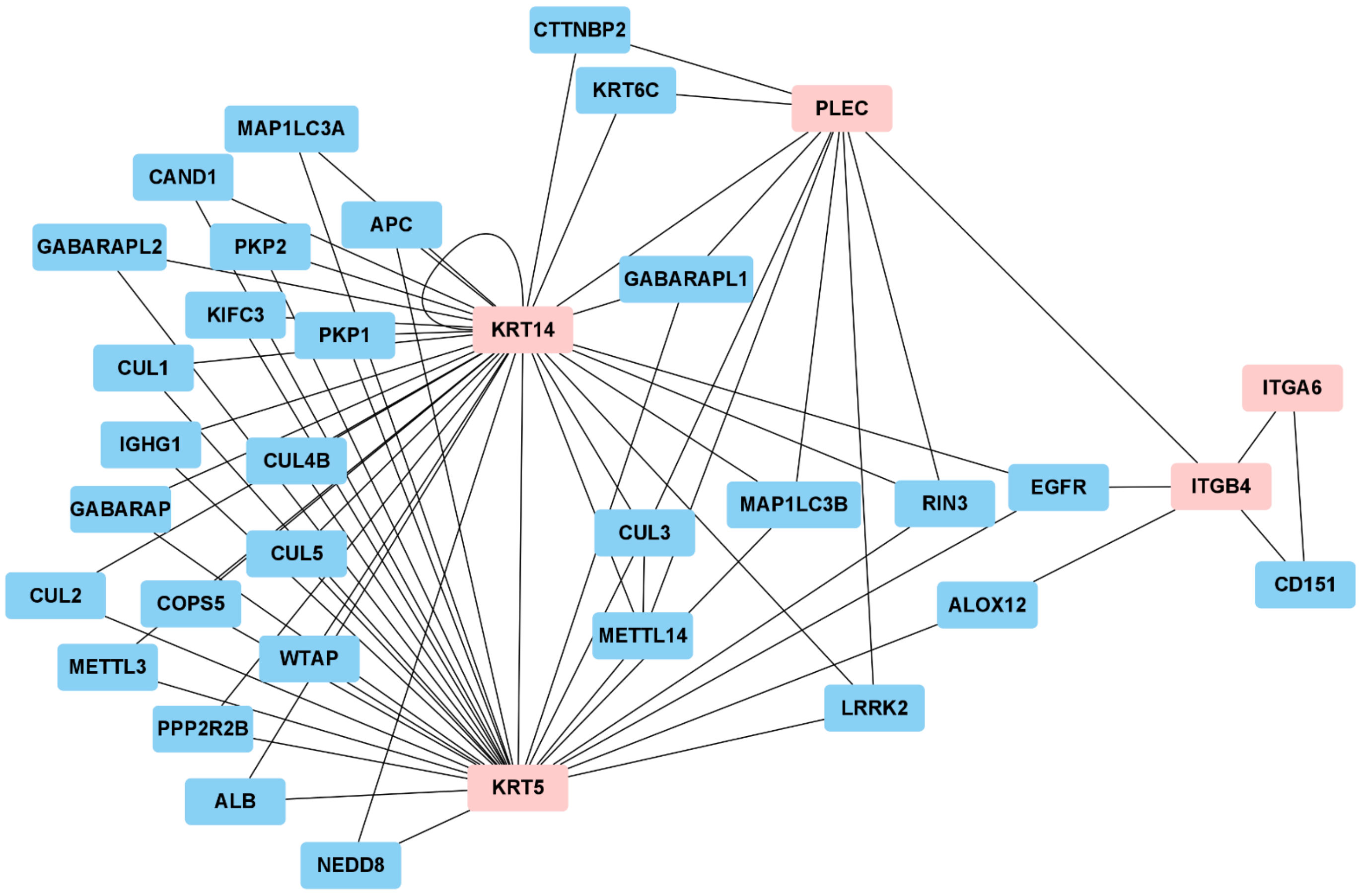

Figure S11. Ribosomal proteins and their direct interactors, experimentally derived using IntAct.

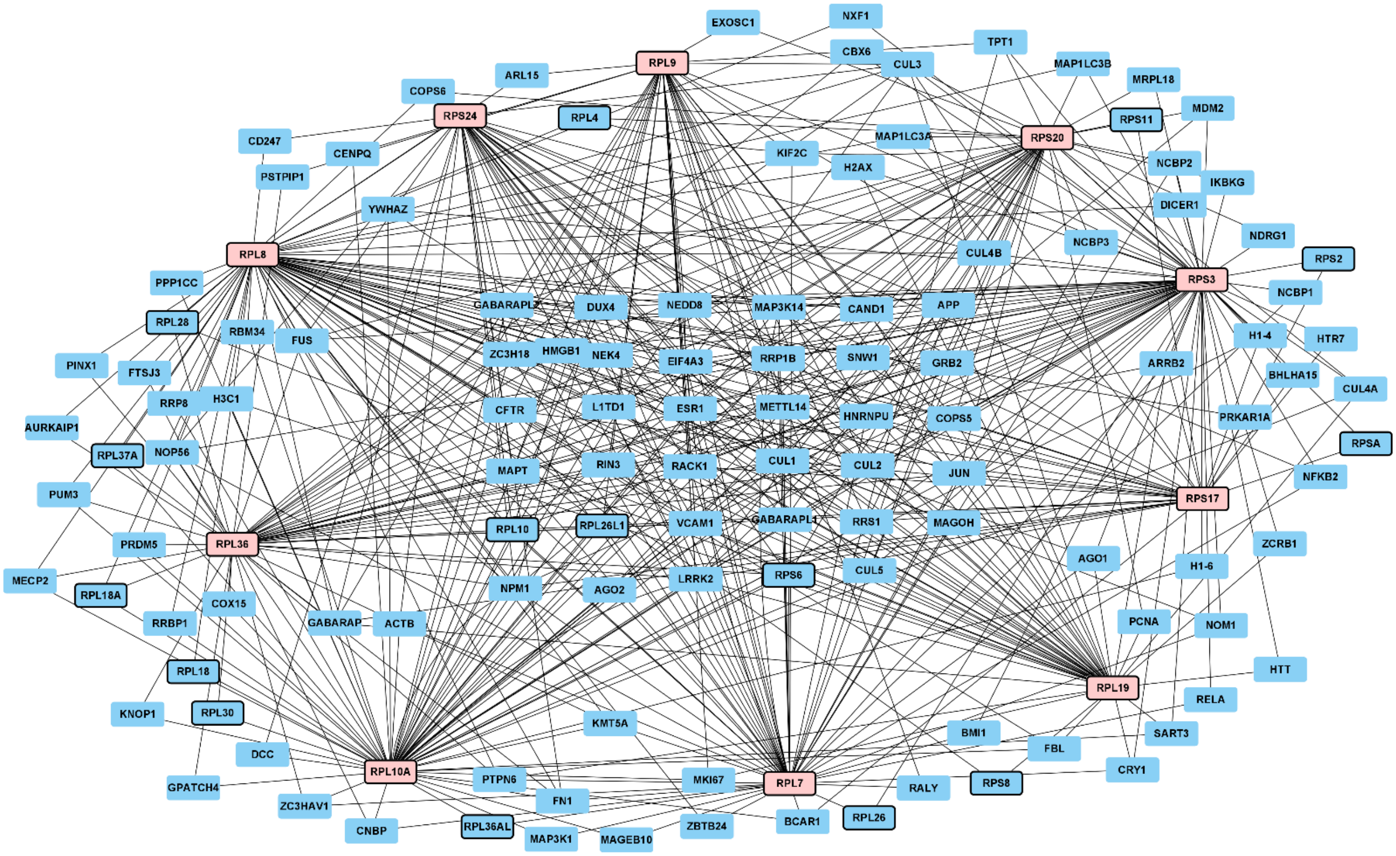

Figure S12. MS1 intensity identifies multiple proteins with significant differences in relative abundance between matched photoaged forearm and intrinsically-aged buttock skin.

ACAD9, ACTN4, ACTR2, ADH5, AHNAK, AIMP1, ALDOA, ANKRD35, ANXA5, ANXA6, ANXA8L1, AP1B1, ARHGDIA, ARPC1B, ARPC5, ATL3, ATP5F1D, ATP6V1E1, B2M, BBOX1, BRK1, CAPG, CAPN1, CAPNS1, CAPNS2, CAPZA2, CBR1, CCDC171, CCT2, CCT3, CCT4, CCT5, CCT7, CD109, CKMT1A, CKMT1B, COX6B1, CRIP2, CRYZ, CSRP1, CTBS, CTNNA1, CYB5A, CYB5R1, CYFIP2, DBT, DCTN2, DKC1, DNAJA1, DSC1, DSC3, DYNC1LI1, DYNLL2, ECHS1, EIF2S3, EIF3D, EIF3G, EIF3I, EIF5A2, ENDOD1, EZR, F2, FADS2, FAM98B, FARSB, FBL, GLUD1, GNAS, GNB1, GOLGA2, GRHPR, GSTP1, H2AFY2, HIST1H2BB, HIST1H3A, HIST1H3B, HIST1H3C, HIST1H3D, HIST1H3E, HIST1H3F, HIST1H3G, HIST1H3H, HIST1H3I, HIST1H3J, HNRNPA2B1, HNRNPH1, HSBP1, HSP90AA1, HSPA8, HSPB1, IARS, IARS2, IDH3G, IGHG3, ILF2, KRT7, KRT79, KRTDAP, LARS, LCP1, LDHB, LETM1, LGALS1, LGALS7, LGALS7B, LIG3, LMNA, LMNB1, MAN2B1, MAPK1, MDH1, METAP1, MVP, MYH13, MYH14, NCCRP1, NDUFA2, NDUFB7, NDUFV2, NPTN, OPA1, PAFAH1B1, PCOLCE, PFN1, PGAM1, PGK1, PKM, PLBD1, PLS3, PPA1, PPA2, PPIA, PPP2CA, PPP2R1A, PRDX3, PRDX4, PRDX5, PRDX6, PRPF19, PSMC4, PSMD11, PYCARD, PYGB, RACK1, RALA, RALY, RAP1A, RARS, RHOT2, RPL10A, RPL12, RPL36, RPL8, RPS12, RPS17, RPS20, RPS7, RTCB, RTN4, RTRAF, RUVBL1, SEPT2, SEPT7, SF3B2, SF3B3, SFN, SH3BGRL, SNRPB, SNRPD2, SNRPF, SNX2, SPTAN1, SPTBN1, SPTBN2, SRP9, SRSF1, SSR1, STAT3, STIP1, SUB1, SYNCRIP, SYTL1, TAGLN, TCP1, TFAM, TIMM10, TIMM13, TPD52, TPM4, TRIM28, TUFT1, TXNL1, URI1, VAMP2, VCP, XRCC5, YWHAB, YWHAE, YWHAG, YWHAZ, ZNF185

ALDH16A1, ALDOC, ALOX12B, APCS, APOD, ARF5, ATP1A2, CAT, CAVIN3, CFD, CNTRL, COL3A1, CSTA, CTNNA2, CTRB1, CTSC, DDX3X, EHD1, EPHX1, FREM1, FRRS1, G3BP1, GLG1, GRN, HDAC1, HIST1H1D, HSP90AB4P, IL36G, IL37, KARS, KATNAL2, KIR2DS1, KRR1, KRT16, KRT31, KRT35, KRT83, KRT84, KRT85, KRT86, KRTAP3-3, KRTAP4-3, LOR, OTOF, PAX7, PFKL, PHOSPHO1, PLXNB2, POTEE, PRKDC, PRSS3P2, PTCHD3, RPL28, RYR1, RYR3, SART1, SCPEP1, SHROOM3, SLC25A13, SMC1A, SNRNP200, SNRPA1, STS, TPP1, TRA2A, TUBAL3, VSTM2L

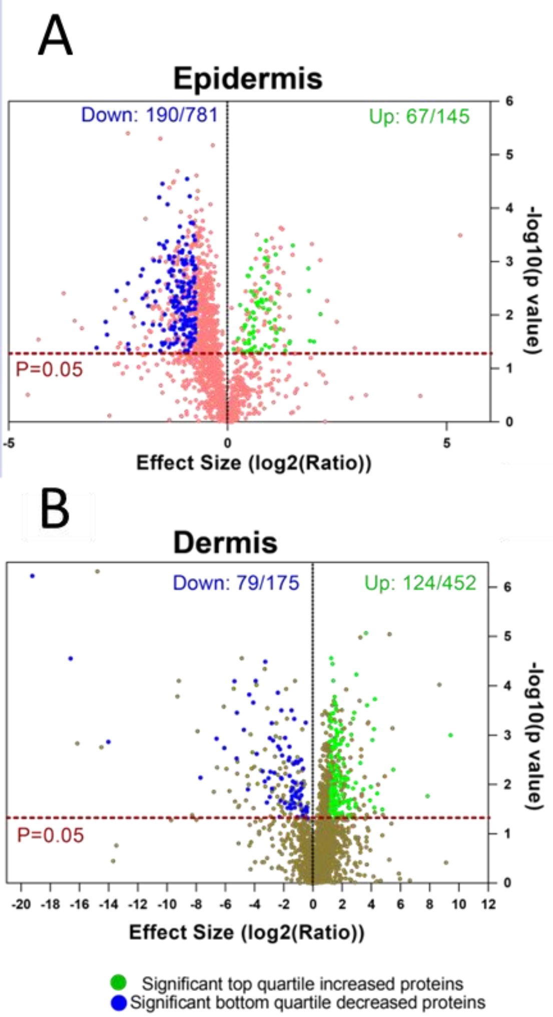

ACO1, ACOT13, ACTN1, ANXA13, AOC2, AOC3, ASPH, ASPM, ASPN, ATP4A, BGN, CALML5, CAMK2A, CEP290, CFD, CHMP1B, COL12A1, COPE, CYFIP1, DCN, DDX4, DPYSL5, DTX3L, DZIP1, DZIP1L, EIF3B, FBH1, FBLL1, FBLN2, FER1L5, GCLM, GGT5, GNA12, GPKOW, HELB, HLCS, HNRNPH1, IGHV3-13, INA, IQCN, LAMB1, LARS, LHX8, LUM, MCU, MTUS2, MYLK, MYO1G, MYOF, NID1, NIPSNAP3A, NSFL1C, OGN, OGT, PCBP2, PDILT, PLIN1, POLR2A, PRELP, RAB14, RAB15, RAB4A, RAB8A, RPN1, RRAS2, SERPIND1, SFRP1, SHC3, SNX2, SPTBN2, SRSF7, SSB, TAGLN, THEGL, TTN, UBA52, UQCR10, USP15, ZNF440

ACP1, ADH1A, AHCY, AIFM1, AK3, ALDH1A1, ALDOA, APCS, APRT, ARPC2, ASPRV1, ATP5F1B, ATP5H, ATP5L, BRK1, C5, CALML3, CCT2, CCT3, CCT5, CCT8, CLEC16A, CLU, CMA1, COL14A1, COL28A1, COMP, COX4I1, COX5A, CTRB1, CXCL12, DERA, DNAJC3, DNAL1, DNM1L, ECH1, EIF3I, EPPK1, EPRS, FADS2, FAM117B, FARSA, FBLN1, FBLN7, FGB, FGL2, FUBP3, GALK1, GLUD2, GM2A, GNAI2, GRN, GSR, GSTK1, HNRNPH2, HPR, HSP90B1, HTRA1, IDE, ITGAV, JUP, KARS, LBP, LFNG, LTBP1, LTBP2, LYVE1, LYZ, MANF, METRNL, MTREX, MXRA5, MYO1D, NARS, NCCRP1, NDUFS1, NDUFS8, NDUFV1, NT5DC3, NTN1, OAF, P3H1, PDHB, PFKL, PGM1, PPA2, PPP2R1A, PRDX2, PRDX3, PRPF19, PSMB6, PSMD11, PYGB, RAB10, RAN, RNASE4, RNPEP, RPS3, RUVBL2, S100A8, S100A9, SCP2, SEC22B, SH3GL1, SNRPF, SOD2, SRI, SRRT, STAM2, SULT2B1, TIMP3, TPP1, TUBA4A, TUBAL3, TUBB2A, TUBB4A, TUFM, TYMP, UQCRC1, VTN, WISP2, XRCC5, ZNF185

Figure S13. Principal component analysis (PCA) with partitioning around medoids clustering (red and green ellipses) of peak ion intensity data used for whole protein relative quantification.

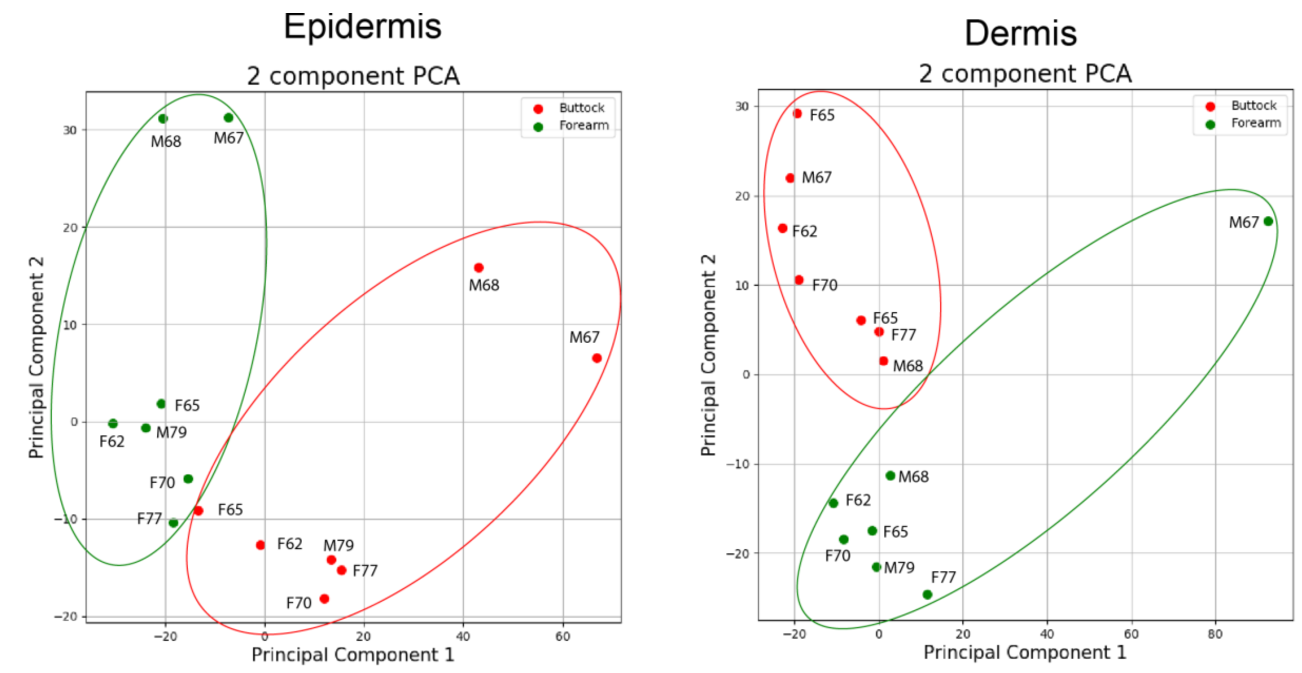

Figure S14. Protein classifications of proteins with significant differences in relative abundance (PANTHER analysis).

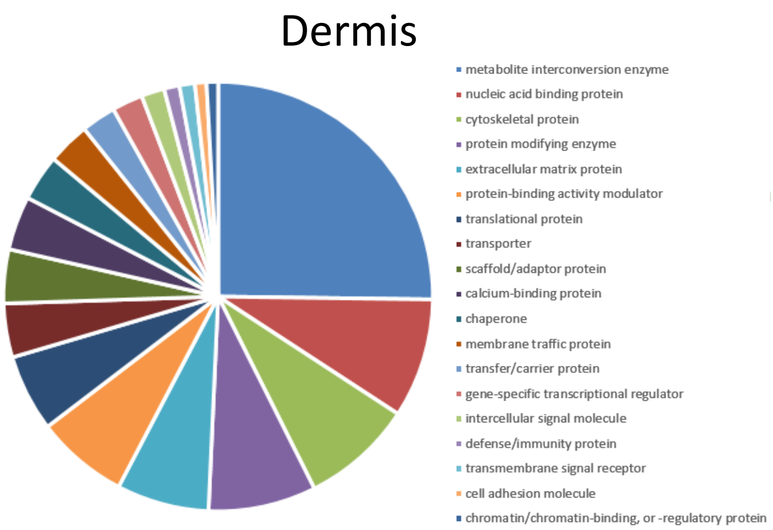

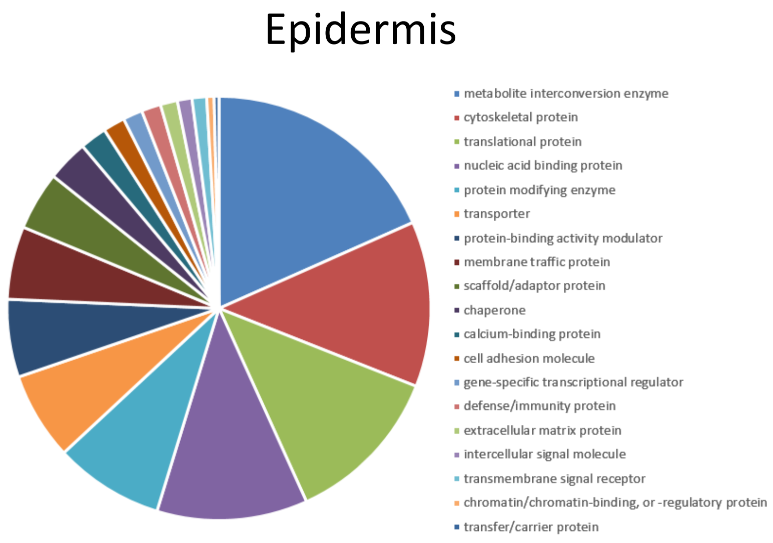

Figure S15. Full western blot membranes for TIMP3 and RPL36 validations.

Dermis

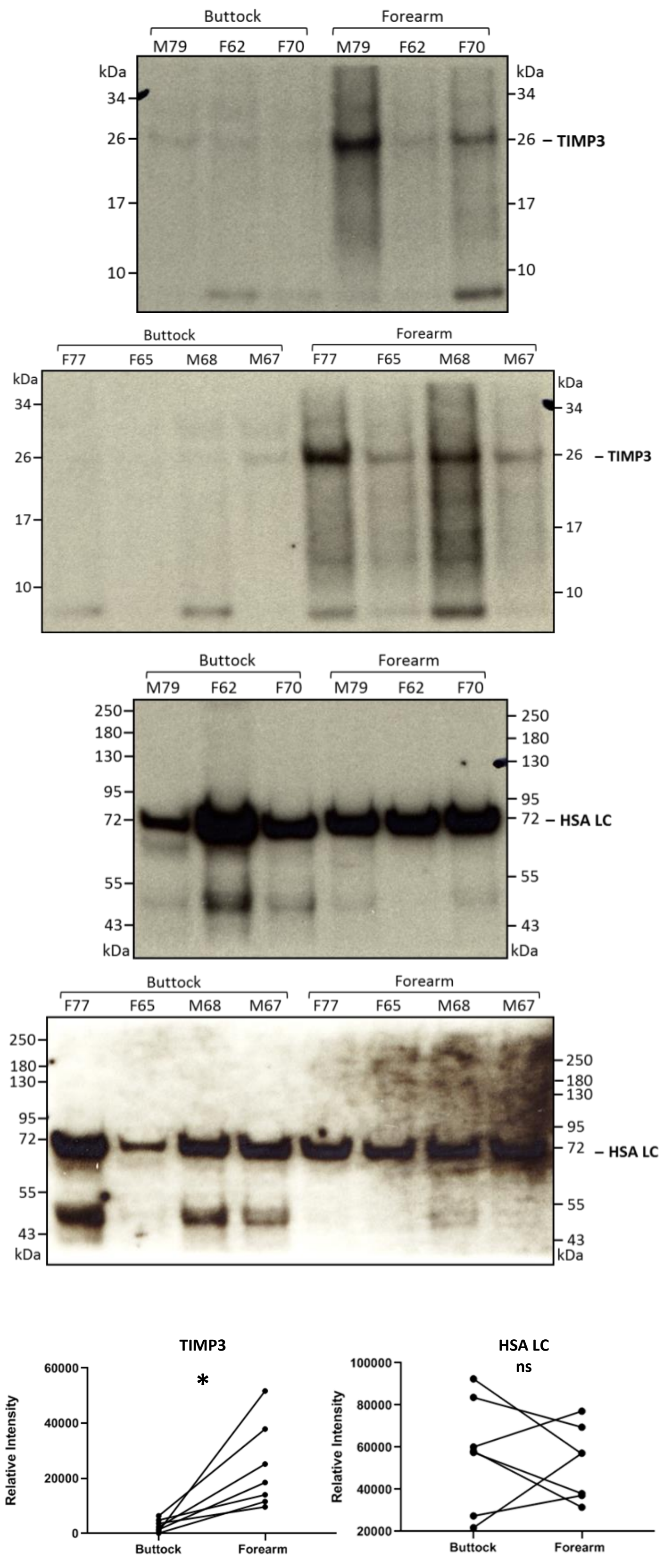

Epidermis

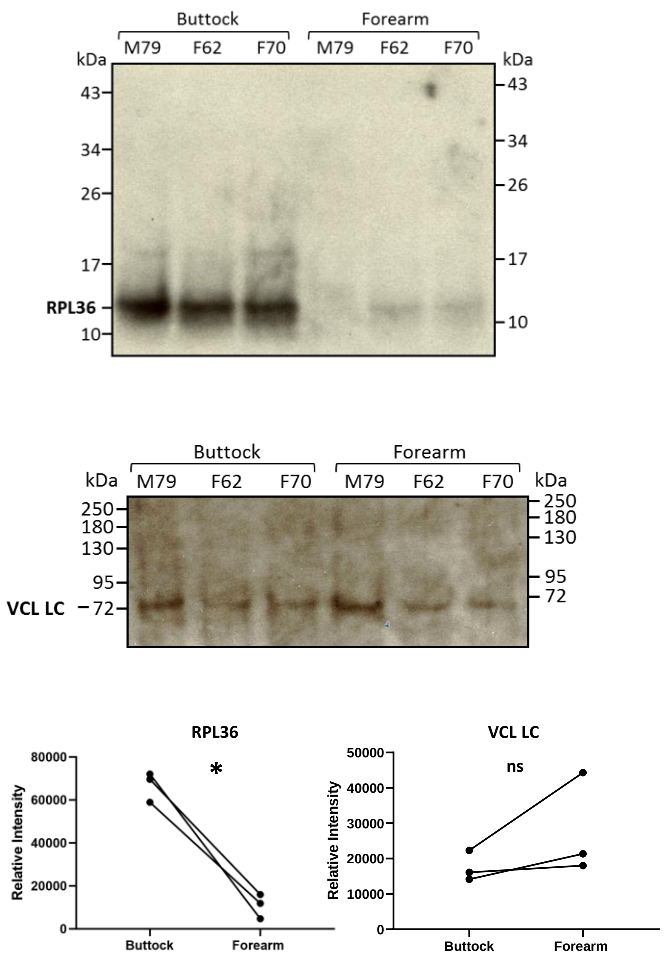

Supplement: Supplementary file 1 — Fig S1‐S15 [file ACEL-20-e13355-s005.pdf]
