# Supplementary material for: Peptide location fingerprinting reveals modification‐associated biomarker candidates of ageing in human tissue proteomes
Source: Aging Cell. 2021 Apr 8;20(5):e13355. doi: 10.1111/acel.13355 (PMC8135079; doi:10.1111/acel.13355)
Supplement: Supplementary file 14 — App S1 [file ACEL-20-e13355-s007.pdf]

Supporting Methods

Tissue cryosectioning and Weigert's staining

Tissue was cryosectioned at a thickness of 5 µm (OTF cryostat; Bright Instruments, Bedfordshire, UK). Three serial sections were collected per slide and stained with Weigert's resorcin fuchsin (Merck; Darmstadt, Germany). Cryosections were fixed in 4% [w/v] paraformaldehyde in PBS and submerged in staining solution, for 10 min per step at room temperature. Stained sections were dehydrated in graded industrial methylated spirit (IMS) (70% [v/v], then twice with 100%), cleared in xylene (5 minutes per step at room temperature) and then mounted (DPX).

Imaging and solar elastosis quantification

Weigert's stained skin sections were imaged using brightfield microscopy (BX53 microscope; Olympus Industrial, Southend-on-Sea, UK). Solar elastosis was quantified by measuring the percentage area of stained elastic fibres using ImageJ (Bethesda, MA, USA). Percentage areas were measured automatically by thresholding the purple stained fibres in the images by brightness using "Threshold Colour". Elastic fibre abundances were measured as a percentage of coloured pixels within the areas. Statistical comparisons were performed using GraphPad Prism (GraphPad Software Incorporated; California, USA). The percentage area of elastic fibres was assessed for matched forearm and buttock skin groups with six individuals per group; measured in three images per section and three sections per individual, and averaged. Elastic fibres were analysed between matched forearm and buttock groups using the Student's paired t-test.

TIMP3 and RPL36 Western blotting

Samples were ran on 4 – 12% NuPAGE gels (Life Technologies, Warrington, UK) and transferred onto Immun-Blot PVDF membranes (Bio-Rad, Watford, UK). Membranes were blocked with 5% (v/v) milk in TBS-T (50 mM Tris-HCl, 5% [v/v] TWEEN 20) for one hour at room temperature and split into two halves along the direction of electrophoresis. Bottom halves of dermal sample membranes were incubated with rabbit anti-TIMP3 (Cell Signalling Technology; D74B10) and top halves with mouse anti-HSA (Abcam, ab10241). Bottom half of epidermal sample membranes were incubated with rabbit anti-RPL36 (Sigma; HPA047153) and top halves with rabbit anti-VCL (Sigma; HPA063777). All primary antibodies were incubated at 1:1000 dilution, overnight at 4ºC. Membranes were washed thrice with TBS-T and

incubated for one hour at room temperature with secondary antibodies (horse radish peroxidase goat anti-mouse or anti-rabbit; Bio-Rad), all at 1:5000 dilution. Blots were developed with Western Lightning Plus ECL (PerkinElmer, Beaconsfield, UK).

Relative quantification of protein abundance with peak area ion intensity using Progenesis QI

Relative quantification of protein abundance was performed using Progenesis QI (Nonlinear Dynamics, Waters, Newcastle, UK). Raw mass spectra files were imported and ion intensity maps were generated. Ion outlines were automatically aligned to a single reference run using default settings. Ion peaks and their relative abundances were then automatically picked without filtering and normalised to a single reference run using default settings. Data were then exported and searched using Mascot v2.5.1 with same search parameters and on the same database as described for peptide location fingerprinting. This was then re-imported back into Progenesis QI where identified peptide ions were matched. Normalised abundance for each protein was calculated as the sum of the each matched peptide ion abundance. Normalised protein abundances, compared between matched forearm and buttock samples, were statistically analysed within Progenesis QI using a paired (repeated measured) ANOVA test. PCA was performed on the quantified proteins using Python Sklearn package **(Fig. S13)**.
