# Supplementary material for: Peptide location fingerprinting reveals modification‐associated biomarker candidates of ageing in human tissue proteomes
Source: Aging Cell. 2021 Apr 8;20(5):e13355. doi: 10.1111/acel.13355 (PMC8135079; doi:10.1111/acel.13355)
Supplement: Supplementary file 15 — App S2 [file ACEL-20-e13355-s014.pdf]

Supporting Figure Legends

Figure S1. Representative histology images of biopsies to visually showcase photoageing phenotype. Weigert's stained cryosections from aged outer forearm skin samples exhibited marked elastosis and disrupted elastic fibre architecture compared to matched buttock samples (sex and age displayed above images).

Figure S2. Skin samples from photoexposed forearm had significant solar elastosis compared to photoprotected buttock. Elastic fibre abundance in forearm skin sections was quantified and compared to that of photoprotected buttock (N = 6, average age = 71 years, range = 65 – 79 years, 3 males; data = median, IQR and range). Elastic fibre abundances in photoexposed forearm skin sections were significantly higher than in photoprotected buttock (p = 0.0012, paired t test).

Figure S3. Principal component analysis (PCA) of spectral count data used for peptide location fingerprinting shows clear separation of forearm and buttock data into distinct clusters. Odd numbered samples corresponded to buttock; even numbered samples corresponded to forearm. Partitioning around medoids clustering analysis automatically separated buttock (red ellipse) and forearm (green ellipse) into two distinct clusters.

Figure S4. Exemplar skin biomarker candidates exhibiting regional differences in peptide yield due to photoageing-specific modifications. Interleaved graph representations of proteins found in **Fig. 3** of main text. Proteins were segmented into 50 amino acid-sized step regions with average peptide counts (PSMs; N = 7) statistically compared between forearm and buttock (graphs = average PSMs, error bars = SD; * ≤ 0.05, ** ≤ 0.01, *** ≤ 0.001, Bonferroni-corrected repeated measures paired ANOVA). Regional significant differences in peptide yield along these protein structures can be seen between photoaged forearm and intrinsically aged buttock.

Figure S5. Exemplar skin biomarker candidates exhibiting regional differences in peptide yield within conserved domains, regions and repeats due to photoageing-specific modifications. Proteins were segmented into step sizes corresponding to conserved domains, repeats or regions pre-defined by the UniProt database with average peptide counts (PSMs; N = 7) heat mapped to each step and compared between forearm and buttock (bar graphs = average PSMs, error bars = SD). Average peptide counts corresponding to each forearm protein step were subtracted from the counts of their corresponding buttock protein step and divided by the aa sequence length of that step to reveal regional differences in peptide yield (line graphs). Multiple protein structures within the dermis and epidermis exhibited statistically significant regional differences in the peptide yield (* ≤ 0.05, ** ≤ 0.01, *** ≤ 0.001; Bonferroni-corrected repeated measures paired ANOVA) between forearm and buttock.

Figure S6. Collagens and their direct experimental interactors. Collagen alpha chains and their complexes (bordered black) were used to build a network of direct, experimental identified interactions curated from MatrixDB. Photoageing biomarker candidates identified with significant regional differences in peptide yield across their structures are shown in pink.

Figure S7. Proteoglycans, glycosaminoglycans and their direct experimental interactors. Proteoglycans and glycosaminoglycans (bordered black) were used to build a network of direct, experimentally identified interactions curated from MatrixDB. Photoageing biomarker candidates identified with significant regional differences in peptide yield across their structures are shown in pink.

Figure S8. TRiC complex subunits (CCTs) and their direct experimental interactors. Chaperonin containing TCPs (CCTs - bordered black) were used to build a network of direct, experimentally identified interactions curated from the IntAct database. Photoageing biomarker candidates identified with significant regional differences in peptide yield across their structures are shown in pink.

Figure S9. Cornification proteins and their direct experimental interactors. Protein entities pertinent to the "formation of the cornified envelope" Reactome pathway were used to build a network of direct, experimentally identified interactions curated from the IntAct database. Photoageing biomarker candidates identified with significant regional differences in peptide yield across their structures are shown in pink. Only proteins with two or more interactions are shown.

Figure S10. Hemidesmosome proteins and their direct experimental interactors. Protein entities pertinent to the “hemidesmosome assembly” Reactome pathway were used to build a network of direct, experimentally identified interactions curated from the IntAct database. Photoageing biomarker candidates identified with significant regional differences in peptide yield across their structures are shown in pink. Only proteins with two or more interactions are shown.

Figure S11. Ribosomal proteins and their direct experimental interactors. Ribosomal proteins (RPL and RPS; bordered black) were used to build a network of direct, experimentally identified interactions curated from the IntAct database. Photoageing biomarker candidates identified with significant regional differences in peptide yield across their structures are shown in pink. Only proteins with two or more interactions are shown.

Figure S12. Label-free relative quantification of protein abundance by peak area ion intensity identifies multiple proteins with significant differences in relative abundance between matched photoaged forearm and intrinsically aged buttock skin. Photoageing had a significant effect on protein abundance in human skin. Proteins with relative abundances which were significantly lower in forearm than in buttock (Progenesis QI multivariate paired ANOVA; $p < 0.05$) and in the bottom quartile for fold change (blue points) are listed to the left of the volcano plots. For epidermis, these include histones, chaperones, heat shock proteins, ribosomal proteins, galectins and redox enzymes. For dermis these include proteoglycans and basement membrane proteins. Proteins with relative abundances which were significantly higher in forearm than in buttock and in the top quartile for fold change (green points) are listed to the right of the volcano plots. For epidermis, this includes a number of keratins and, for dermis, a number of collagens, protease modulators and elastic fibre proteins.

Figure S13. PCA analysis of peak area ion intensity data used for relative quantification shows clear data separation between forearm and buttock samples analysed. Partitioning around medoids clustering analysis automatically separated buttock (red) and forearm (green) into two distinct clusters.

Figure S14. Classification of protein biomarker candidates significantly different in relative abundance into functional groups reveals metabolite interconversion enzymes, nucleic-acid binding proteins, cytoskeletal proteins and translational proteins as the main classes in skin most affected by the photoageing process. Biomarker candidate proteins with significant differences in abundance between forearm and buttock according to peak area ion intensity analysis were categorised into protein classes (PANTHER classification system; large multi-coloured pie charts: clockwise rankings with top rank at 12:00; only classes with two or more proteins are represented). Metabolite interconversion enzymes, nucleic acid-binding proteins and cytoskeletal proteins were in the top four classes affected for both dermis and epidermis. Protein modifying enzymes for dermis and translational proteins for epidermis were also in the top four for each respective sub tissue. Although the majority of the dermis is comprised of ECM, ECM proteins were not in the top four classes affected. This indicates that relative protein abundance measurements are not as suitable as protein modification measurements by peptide location fingerprinting (**Fig. 4** in main text) at distinguishing photoageing-related differences to ECM proteins.

Figure S15. Western blot validation of LC-MS/MS relative quantification of protein abundance highlights TIMP3 and RPL36 as novel biomarker candidates of photoageing. Relative abundance measurements of two proteins identified as significantly different between matched photoaged forearm and intrinsically aged buttock samples by peak area ion intensity was confirmed by Western blotting. TIMP3 detection was negligible in buttock dermis samples from aged individuals (sex and age in years labelled per lane) but highly present in matched forearm dermis samples (loading control [LC] = human serum albumin – HSA). Quantification of relative intensity shows that TIMP3 abundance is significantly higher in forearm than in matched buttock (paired t test, $p = 0.0115$). In contrast, RPL36 was highly present in buttock epidermis samples from aged individuals but negligibly detected in matched forearm epidermis samples (LC = vinculin, VCL). Quantification of relative intensity shows that RPL36 abundance is significantly lower in forearm than in matched buttock (paired t test, $p = 0.0112$). LCs (HSA for dermis samples and VCL for epidermis samples) were chosen based on their lack of significant differences in relative abundance between forearm and buttock samples according to LC-MS/MS peak area ion intensity. Western blot analysis of these controls showed no significant differences between forearm and buttock samples also corroborates peak area intensity comparisons.
